# VvD14c-VvMAX2-VvLOB/VvLBD19 module is involved in the strigolactone-mediated regulation of grapevine root architecture

**DOI:** 10.1186/s43897-024-00117-z

**Published:** 2024-10-25

**Authors:** Yan Xu, Zhengxin Lv, Muhammad Aamir Manzoor, Linhong Song, Maosen Wang, Lei Wang, Shiping Wang, Caixi Zhang, Songtao Jiu

**Affiliations:** Department of Plant Science, School of Agriculture and Biology, Shanghai Jiao Tong University, Dongchuan Road No. 800, Shanghai, 200240 P. R. China

**Keywords:** Grapevines, Strigolactones, Root architecture, *DWARF 14*, *MORE AXILLARY GROWTH 2*

## Abstract

The D14 protein, an alpha/beta hydrolase, is a key receptor in the strigolactone (SL) signaling pathway. However, the response of VvD14 to SL signals and its role in grapevine root architecture formation remain unclear. This study demonstrated that *VvD14c* was highly expressed in grapevine tissues and fruit stages than other *VvD14* isoforms. Application of GR24, an SL analog, enhanced the elongation and diameter of adventitious roots but inhibited the elongation and density of lateral roots (LRs) and increased *VvD14c* expression. Additionally, GR24 is nested within the VvD14c pocket and strongly bound to the VvD14c protein, with an affinity of 5.65 × 10^−9 ^M. Furthermore, VvD14c interacted with grapevine MORE AXILLARY GROWTH 2 (VvMAX2) in a GR24-dependent manner. Overexpression of *VvD14c* in the *d14* mutant and *VvMAX2* in the *max2 Arabidopsis* mutant reversed the increased LR number and density, as well as primary root elongation. Conversely, homologous overexpression of *VvD14c* and *VvMAX2* resulted in reduced LR number and density in grapevines. VvMAX2 directly interacted with LATERAL ORGAN BOUNDARY (VvLOB) and VvLBD19, thereby positively regulating LR density. These findings highlight the role of SLs in regulating grapevine root architecture, potentially via the VvD14c-VvMAX2-VvLOB/VvLBD19 module, providing new insights into the regulation of root growth and development in grapevines.

**Supplementary Information:**

The online version contains supplementary material available at 10.1186/s43897-024-00117-z.

## Core

GR24 significantly enhanced the elongation and diameter of ARs, but inhibited the elongation and density of LRs, and increased *VvD14c* expression in grapevines. GR24 is nested in the pocket of VvD14c and surrounded by its residues. VvD14c protein strongly bound to GR24 with an affinity of 5.65 × 10^−9 ^M. VvD14c interacts with VvMAX2 in a GR24-dependent manner. Both VvD14c and VvMAX2 are crucial for regulating grapevine root architecture. VvMAX2 directly interacts with VvLOB and VvLBD19, positively regulating grapevine LR density.

## Gene and accession numbers

The sequence data are available on the National Center for Biotechnology Information (NCBI) website (https://www.ncbi.nlm.nih.gov/).

## Introduction

The root system of a plant comprises primary, lateral, and adventitious roots (ARs), all of which share the same radial tissue organization (Schiefelbein and Benfey 1991). The final architecture of the root system is significantly influenced by the growth rate of the primary root as well as the location, spacing, and growth rate of lateral roots (Ingram and Malamy 2010). Strigolactones (SLs), a class of terpenoid lactones, are initially identified as root-derived signals that stimulate the germination of parasitic plants, particularly Orobanche and Striga (Cook et al. 1966; Bouwmeester et al. 2003; Humphrey and Beale 2006). Furthermore, SLs are endogenous hormones that play a crucial role in regulating plant growth and development (Umehara et al. 2008; Gomez-Roldan et al. 2008). They can inhibit lateral bud growth primarily by suppressing cytokinin synthesis or promoting auxin biosynthesis (Brewer et al. 2009; Johnson et al. 2006). SLs and related signaling events also influence various aspects of root growth and development, including primary root (PR) elongation, AR initiation, lateral root (LR) development and growth, and root hair (RH) formation and elongation in numerous plant species (Sun et al. 2022). For instance, under normal conditions, SLs promote seminal root elongation in rice and PR and RH elongation in *Arabidopsis*. However, they inhibit LR development in *Arabidopsis*, rice, and peas. The effects of SLs on AR elongation and development in plants vary depending on the species and growth stage. In *Arabidopsis* and other eudicot plants, SLs inhibit AR formation, while in gramineous plants such as rice and maize, they increase AR elongation and quantity (Sun et al. 2022). These physiological processes are essential for water and nutrients absorption from the soil (Koltai et al. 2010; Arite et al. 2012).

D14, an alpha/beta (α/β)-hydrolase receptor, possesses both receptor and hydrolase functions (Seto et al. 2019; Yao et al. 2016). In a previous study, overexpression of *D14D* in *Gossypium hirsutum* seedlings rescued the *d14* mutant phenotype, which includes reduced shoot branching, decreased rosette leaf length-width ratio, and shorter plant height (Zhu et al. 2021). Although *Populus* has two *PtD14* genes, overexpression of only *PtD14a* can restore the phenotype of the *Atd14* mutant (Zheng et al. 2016). The interaction of AtD14/D14 (*Arabidopsis*/rice) protein with SLs induces a conformational change in AtD14/D14, leading to its binding with the F-box protein MAX2/D3 in both *Arabidopsis* and rice (Sun et al. 2022). This interaction facilitates the assembly of the SCF^MAX2^ ubiquitin ligase complex, which comprises Skp1, Cullin, and F-box proteins. The SCF^MAX2^ complex targets the class I Clp-ATPase suppressor D53 in rice and SUPPRESSOR OF MAX2-LIKE 6, 7, and 8 (*SMXL6*,* 7*, and *8*) in *Arabidopsis* for degradation via the polyubiquitin/proteasome pathway (Wang et al. 2020a; Zhou et al. 2015). Rice D53 and *Arabidopsis* SMXL6, 7, and 8 engage in protein-protein interactions with TOPLESS-related (TPR) and TOPLESS (TPL) proteins. *SMXL6*,* 7*, and *8*, along with *TPR2*, exhibit enhanced transcriptional inhibitory activities, further facilitating their suppressive functions (Wang et al. 2015). Wang et al. (2020a) demonstrated that SMXL6 acts as a transcription factor, directly inhibiting the transcription of SMXL6, 7, and 8. Moreover, SMXL6 can form complexes with unknown transcription factors, suppressing the expression of downstream genes such as PRODUCTION OF ANTHOCYANIN PIGMENT 1 (*PAP1)* and BRANCHED 1 (*BRC1*). The degradation of SMXL6, 7, and 8 alleviates the inhibitory effects on the expression of proteins encoded by these downstream genes. In addition to governing plant growth and development, these proteins also play a role in the full activation of stress responses (Wang et al. 2015, 2020a). However, the SL signal transduction pathway and the functions of key genes, including *VvD14* and *VvMAX2*, in grapevine root architecture remain to be elucidated.

Root development is associated with the COBRA (COB), GRAS, and LOB domain gene families (Frank and Roman 2008). This association is particularly prominent within the LOB DOMAIN/ASYMMETRIC LEAVES2-LIKE (LBD/ASL) gene family, which encodes a conserved, plant-specific transcription factor class distinct from its LOB domain (Sun et al. 2013). The LBD/ASL gene family influences leaf polarity establishment (Xu et al. 2003), LR formation (Jungmook and Han 2014), boundary delimitation (Borghi et al. 2007), tracheary element development (Soyano et al. 2008), female gametophyte development (Evans 2007), cytokinin signaling (Naito et al. 2007), inflorescence branch formation (Bortiri et al. 2006), in vitro regeneration (Xu et al. 2018), and regulation of the expression of *KNOX* genes (Borghi et al. 2007). Based on its expression pattern, it has been postulated that LOB plays a role in the establishment of boundaries between the meristem and differentiated lateral organs (Shuai, 2002). Additionally, AtLBD19 is a transcription factor involved in regulating root development in plants (Liu et al. 2019). LBD18 is a key regulator of LRs. During the auxin response, it synergizes with LBD16 or LBD29 downstream of ARF7 and ARF19. Furthermore, LBD18 binds directly to the *EXP14* promoter, thereby activating *EXP14*, which encodes a cell wall-loosening factor. This interaction facilitates the emergence of LRs (Jungmook and Han 2014). Notably, rice *Crl1* (*Arl1*) and maize *Rtcs* (Liu et al. 2005; Taramino et al. 2007), which are closely related to *AtASL16* (*AtLBD29*), play critical roles in crown root formation in monocots. However, to our knowledge, no studies have investigated the role of the association between LBD family members and SLs in the regulation of root architecture.

This study investigated the activation of *VvD14c* by exogenous GR24 treatment, revealing its high expression levels across various grapevine tissues and fruit developmental stages. Molecular docking simulations demonstrated that GR24 nestles within the binding pocket of VvD14c, forming specific hydrogen bonds and hydrophobic interactions with the surrounding residues. In addition, the VvD14c protein was found to strongly bind to GR24 with an affinity of 5.65 ×10^−9 ^M. Furthermore, the biological functions of *VvD14c* and its interacting protein *VvMAX2* were elucidated. The study also established that VvMAX2 directly interacts with VvLOB and VvLBD19, which positively regulate LR density in grapevines. These findings provide a foundation for future investigations into the role of SLs in modulating grapevine root architecture.

## Results

### Expression, sequence and phylogenetic analyses, and subcellular localization of *VvD14c*

**Fig. 1:**
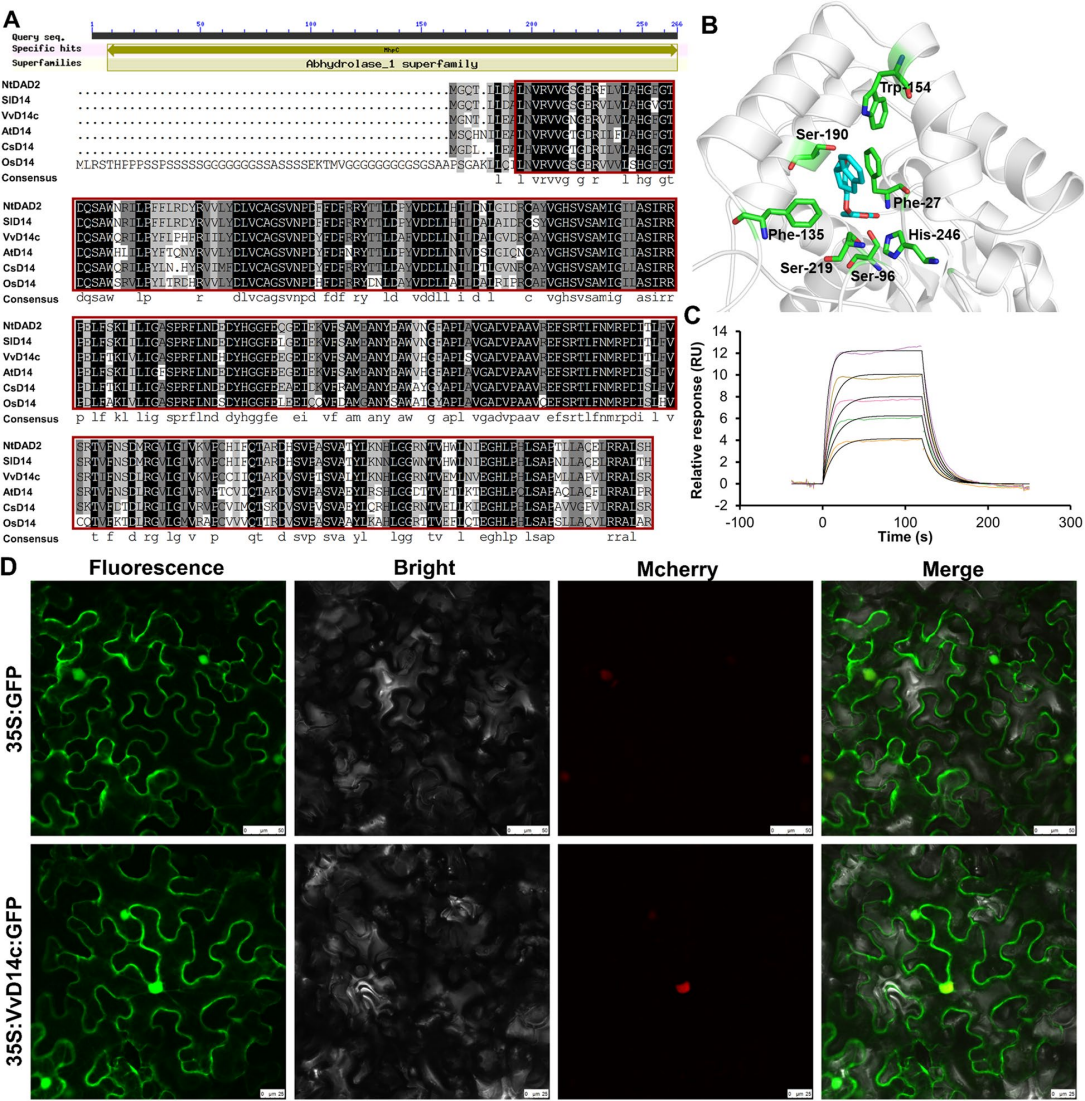
Phylogenetic tree, sequence analysis, and subcellular localization of VvD14c.** A** Multiple sequence alignment of VvD14c. **B** Molecular recognition of GR24 by VvD14c. Location of the pocket residues (green) whose mutations compromised GR24 binding to VvD14c. **C** VvD14c protein strongly bound to GR24. **D** Subcellular localization of VvD14c. The 35S: GFP was used as the positive control. Green fluorescence represents the green fluorescent protein (GFP) fusion protein signal. Red fluorescence represents the nucleus marker p2300-mCherry. Yellow indicates merged signals. NtDAD2 (XP_019258478), SlD14 (XP_004238093), VvD14c (XP_010664515), AtD14 (NP_566220), CsD14 (XP_006472297), and OsD14 (NP_001404251)

To elucidate the function of *VvD14* genes in grapevine, we investigated their tissue-specific and spatiotemporal expression patterns. Semi-quantitative and quantitative PCR assays demonstrated that *VvD14c* exhibited higher expression levels than other *VvD14* members across various tissues and fruit developmental stages of grapevines (Additional File 1: Fig. S1). This indicates that *VvD14c* is a primary gene within the *D14* family involved in grapevine growth and development. Accordingly, we selected *VvD14c* as the target gene to clarify its function in grapevines. *VvD14c* encodes a 267-amino-acid protein and is a member of the α/β hydrolase family (Fig. 1A). Molecular docking results indicated that GR24 is nested within the VvD14c pocket, surrounded by residues such as Ser190, Phe135, His246, and Phe27, forming specific hydrogen bonds and hydrophobic interactions (Fig. 1B). In addition, the VvD14c protein strongly bound to GR24 with an affinity of 5.65 × 10^−9^M (Fig. 1C). Phylogenetic analysis revealed that *NtDAD2* from *Nicotiana tabacum* clustered with *SlD14* from *Solanum lycopersicum*, both of which are closely related to *VvD14c*, suggesting comparable functions among them (Additional File 1: Fig. S2.). Fluorescence microscopy demonstrated that the 35S:VvD14c:GFP fusion proteins were present exclusively in the cell nucleus and cytoplasm, indicating their functional localization (Fig. 1D).

### Exogenous GR24 treatment modulates root architecture in grapevine

**Fig. 2:**
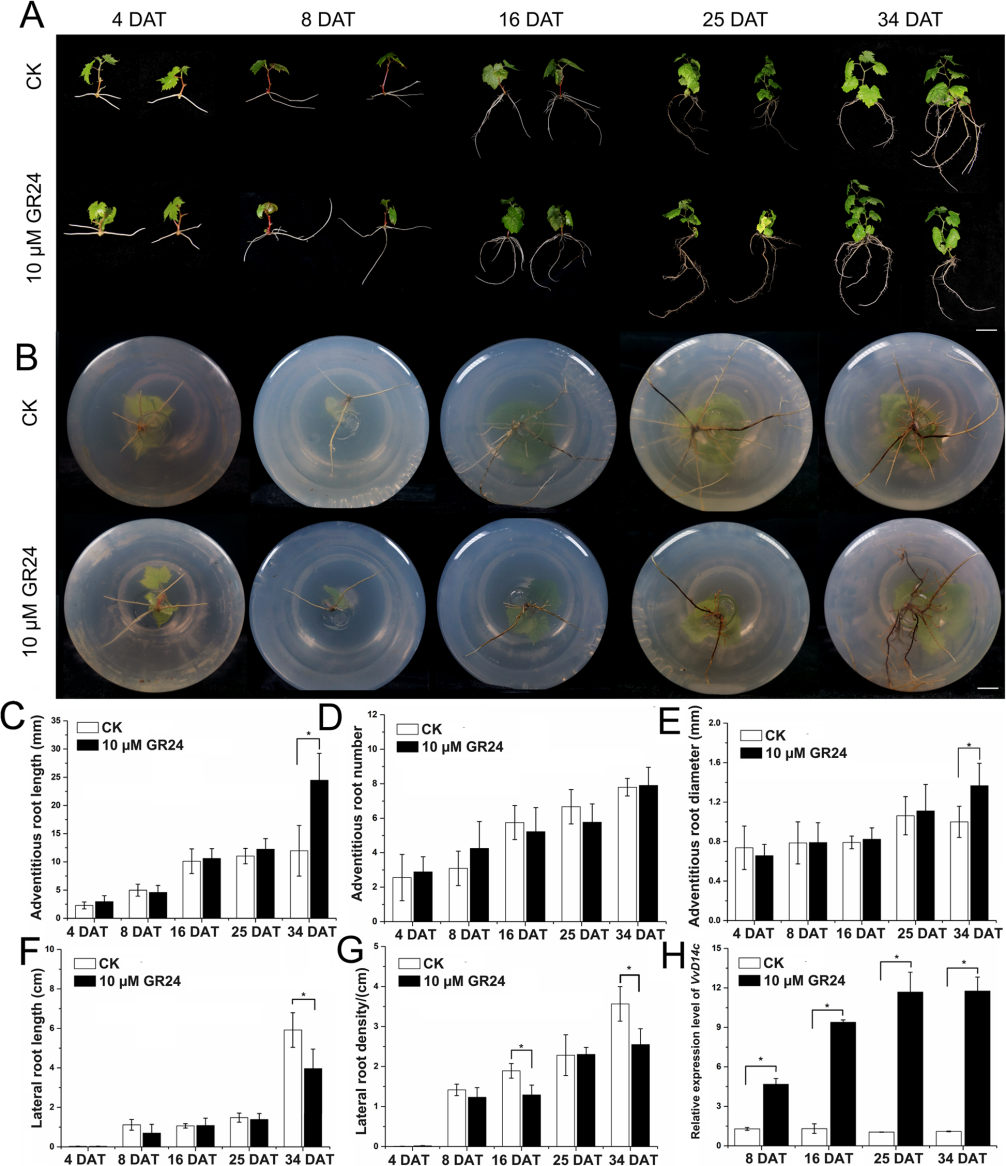
Strigolactones (SLs) regulate adventitious and lateral root growth and development of grapevine. **A**,** B** Phenotypes of roots and whole grapevine seedlings (*Vitis vinifera* cv. ‘Thompson Seedless’) treated with GR24, a synthetic SL analog. Roots were collected from seedlings with similar stem heights at 4, 8, 16, 25, and 34 days after treatment (DAT) and flash-frozen using liquid nitrogen. Scale bar: 2 cm. **C–G** Changes in the length (**C**), number (**D**), and diameter (**E**) of ARs, lateral root length (**F**), and density (**G**) under GR24 treatment at 4, 8, 16, 25, and 34 DAT. Data in (**C–G**) are presented as the mean ± standard deviation. CK, control. **H** The expression levels of *VvD14c*, a SL receptor gene, under GR24 treatment at 8, 16, 25, and 34 DAT. Data in (**H**) are the mean ± standard deviation of values obtained from two treatments from at least three biological replicates. Significant differences were assessed using Student’s *t*-test (*p* < 0.05)

To clarify the role of SLs in root development, grapevine plantlets were treated with 10 µM GR24, a synthetic SL analog. The phenotypes of ARs and LRs were observed at 4, 8, 16, 25, and 34 days after treatment (DAT) (Fig. 2). At 34 DAT, GR24 treatment promoted the elongation and thickening of ARs compared with those in the control group (Fig. 2C and E). In contrast, GR24 treatment significantly inhibited LR elongation and density at 34 DAT (Fig. 2F and G). Following treatment with 10 µM GR24, *VvD14c*, a putative SL receptor gene, was significantly upregulated in the roots at 8, 16, 25, and 34 DAT (Fig. 2H). Collectively, these results demonstrate that SLs play a crucial role in regulating the root architecture of grapevines.

### Roles of *VvD14c* in regulating grapevine root architecture

**Fig. 3:**
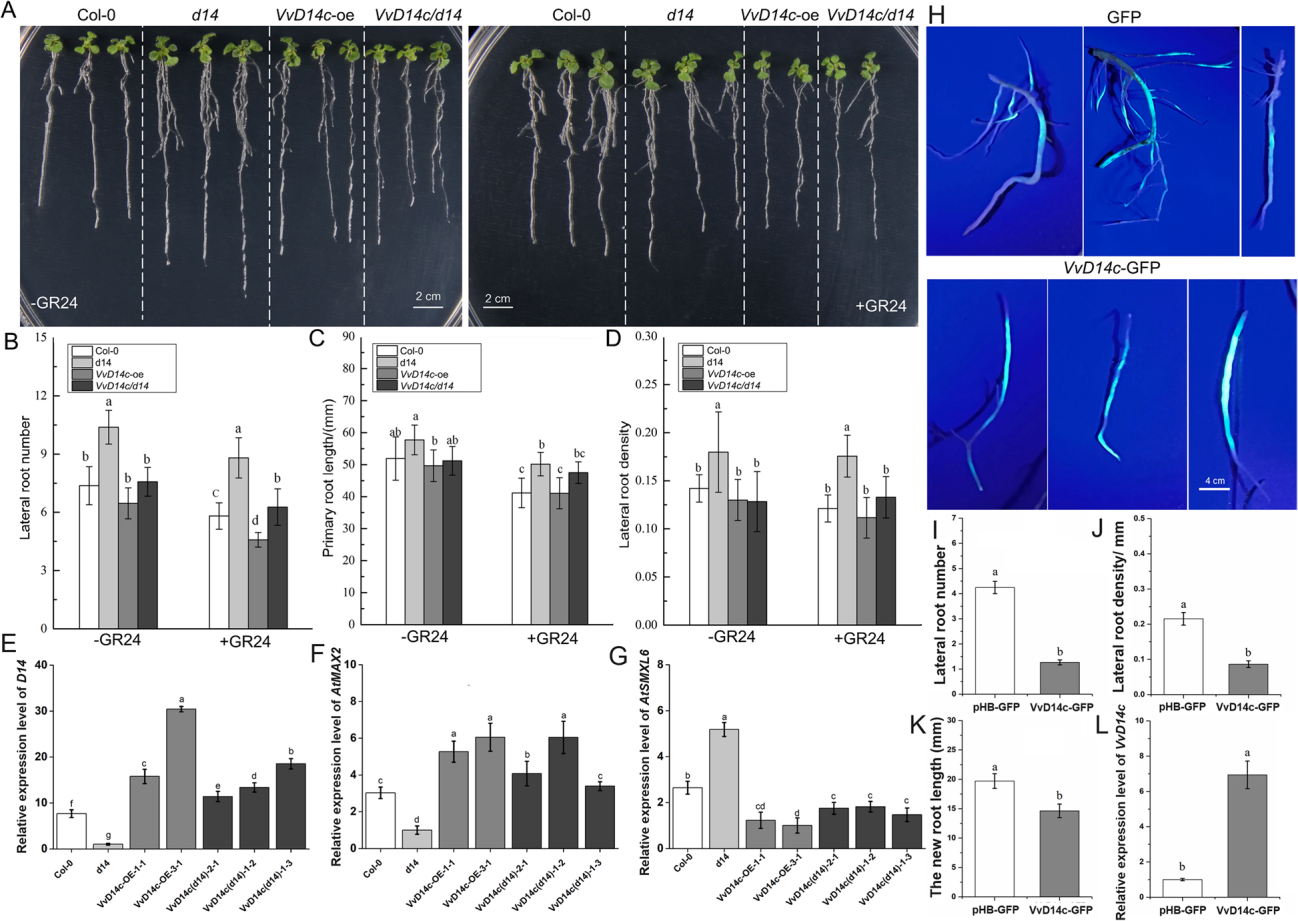
*VvD14c* negatively regulates lateral root number and density.** A** Phenotypes of Col-0, *d14*, VvD14c-OE, and VvD14C/d14 roots grown on 1/2 MS medium with (right) or without (left) 5 µM GR24. Scale bar, 2 cm. **B–D** Changes in lateral root number (**B**), primary root length (**C**), and lateral root density (**D**) in Col-0, *d14*, VvD14c-OE, and VvD14C/d14 seedlings grown on media with and without GR24. Data in (**B–D**) represent the mean ± standard deviation (SD) from at least 50 biological repetitions. **E–G** Expression of *D14* (**E**), *AtMAX2* (**F**), and *AtSMXL6* (**G**) was analyzed using reverse transcription-quantitative polymerase chain reaction (RT-qPCR) in Col-0, *d14*, VvD14c-OE, and VvD14C/d14 roots. Data are the mean ± SD of values from at least three biological repetitions. Significant differences were assessed using Student’s *t*-test (*p < 0.05). **H** Phenotypes of green fluorescent protein (GFP) and *VvD14c*-OE hairy roots after 80 days of *Agrobacterium rhizogenes* infection. Scale bar, 4 cm. **I–K** Changes in the lateral root number (**I**), density (**J**), and new root length (**K**) in GFP and *VvD14c*-OE hairy roots. Data in (**I–K**) represent the mean ± SD of values from at least 30 biological repetitions. **L** Expression of *VvD14c* was detected using RT-qPCR in GFP and *VvD14c*-OE hairy roots. Data in (**L**) represent the mean ± SD of values from three biological repetitions. Significant differences were assessed using Student’s *t*-test (*p < 0.05)

Compared to the control plants, i.e., Columbia 0 (Col-0) plantlets, the *d14 Arabidopsis* mutants exhibited high number and density of LRs as well as long PRs when treated with 5 µM GR24 (Fig. 3A). Without the 5 µM GR24 treatment, *VvD14c/d14* (overexpressing VvD14c in the *d14* mutant) plants showed no significant differences in LR number, density, or PR length when compared with Col-0 plantlets (Fig. 3B-D). These results indicated that *VvD14c* overexpression reversed the root growth and development phenotype of the *d14 Arabidopsis* mutant, suggesting that *VvD14c* negatively regulates LR number and density. Additionally, the *d14* mutant exhibited smaller plants with narrower and shorter leaves than those of Col-0 plants (Additional File 1: Fig. S3). However, no significant differences were observed in leaf length and width, and petiole length between the *VvD14c/d14* and Col-0 plants, indicating that *VvD14c* overexpression also restored the aboveground phenotype of the *d14 Arabidopsis* mutant. These findings demonstrate that *VvD14c* possesses biological functions similar to those of *d14* *Arabidopsis*. The expression of SL signaling-related genes, such as *D14*, *AtMAX2*, and *AtSMXL6*, was also investigated in transgenic seedlings (Fig. 3E-G). Compared with that in Col-0 plants, the expression of *D14* and *AtMAX2* was significantly inhibited in the *Arabidopsis d14* mutant but significantly increased in VvD14c-overexpressing (VvD14c-OE) plants. In *VvD14c*/*d14* plants, the expression of *D14* and *AtMAX2* was increased compared with that in Col-0 plants. Conversely, *AtSMXL6* expression was markedly induced in the *d14* mutant and notably reduced in the *VvD14c*-OE plants. Overexpression of *VvD14c* resulted in reduced number and density of LRs in grapevines (Fig. 3H–L). Furthermore, *VvD14c* expression was significantly higher in *VvD14c*-OE hairy roots than in GFP-empty vector roots.

### VvD14c interacts with VvMAX2 in a GR24-dependent manner

**Fig. 4:**
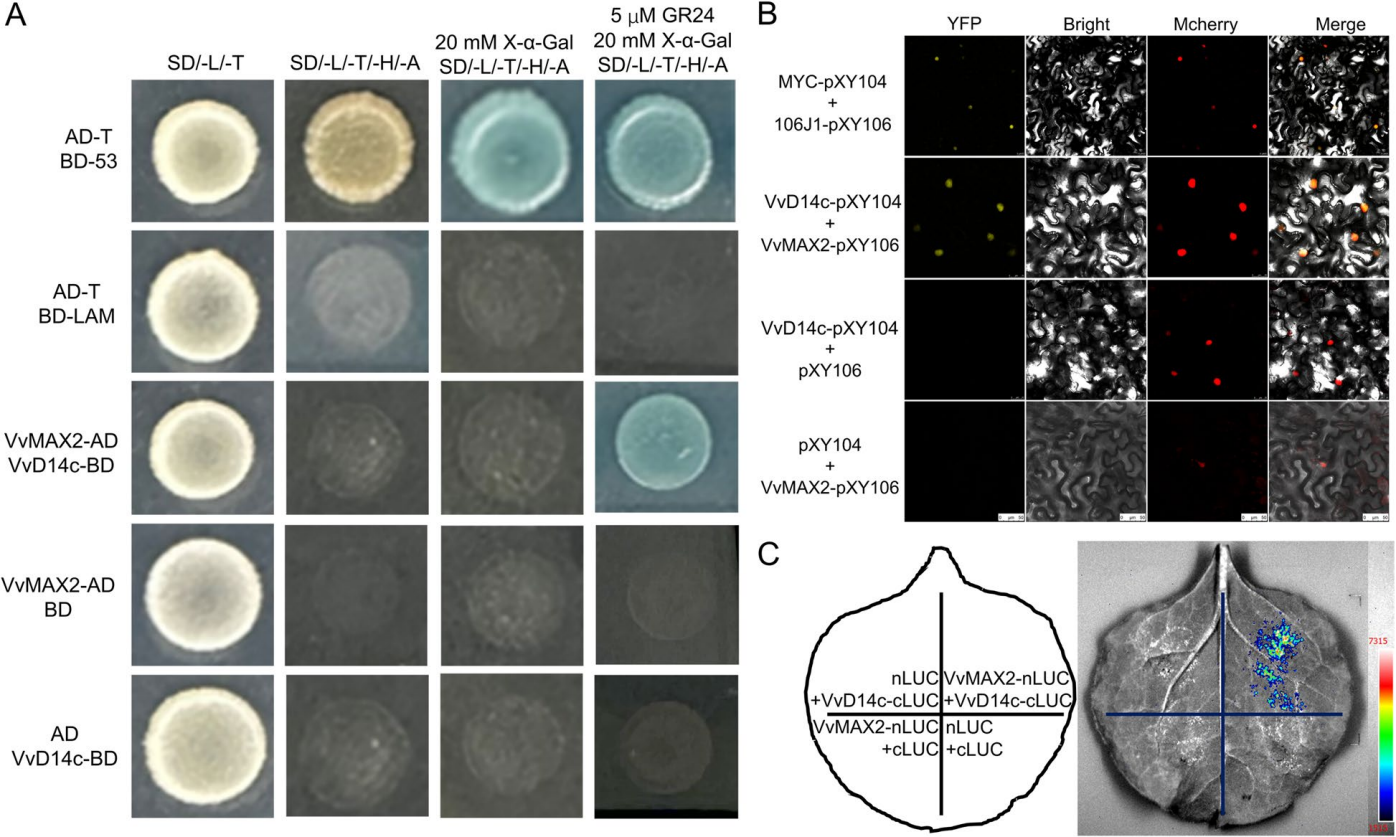
*VvD14c* physically interacts with *VvMAX2* in a GR24-dependent manner in vivo. **A** Yeast two-hybrid assay confirmed the interaction between VvD14c and VvMAX2, contingent upon the presence of GR24. Yeast cells were cultured on SD/-Leu/-Trp medium for 2 days or on SD-Leu/-Trp/-Ade/-His medium supplemented with either 5 µM GR24 or 20 mM X-α-gal for 5 days. **B** BiFC assay demonstrated the interaction between VvD14c and VvMAX2. Full-length VvD14c was fused with the split N-terminal (nYFP) fragment, while VvMAX2 was fused with the split C-terminal (cYFP) fragments of YFP. Bars: 25 μm. p2300-mCherry served as the nuclear marker. **C** Split luciferase complementation assay (SLCA) further validated the interaction of VvMAX2 with VvD14c. VvMAX2-nLUC and VvD14c-cLUC constructs were used to co-transformed into tobacco leaves, and the LUC signal was detected after 48 h. Representative images are depicted in the left panel, and the corresponding luciferase activity is quantified in the right panel

To further elucidate the function of VvD14c, we conducted a yeast two-hybrid (Y2H) assay to identify proteins interacting with VvD14c. We individually fused *VvD14c* (LOC100257558) and *VvMAX2* (LOC104880827) with the pGBKT7 (BD) and AD vectors (Fig. 4A). In the presence of 5 µM GR24 and 20 mM X-α-Gal, yeast cells harboring BD-VvD14c and AD-VvMAX2 exhibited a dark blue color, indicating a GR24-dependent interaction between VvD14c and VvMAX2 (Fig. 4A). We further corroborated the interaction between VvD14c and VvMAX2 using an in vivo bimolecular fluorescence complementation (BiFC) assay in tobacco leaves. Co-expression of VvD14c-cYFP and nYFP-VvMAX2 resulted in a strong YFP signal in tobacco cells. In contrast, no YFP signal was detected when they were co-expressed with nYFP and VvD14c-cYFP or cYFP and nYFP-VvMAX2, confirming a positive interaction between VvD14c and VvMAX2 (Fig. 4B). To provide additional evidence for the interaction between VvMAX2 and VvD14c, we performed a split-luciferase complementation assay (SLCA) using VvMAX2 fused with N-terminal luciferase (VvMAX2-nLUC) and VvD14c fused with C-terminal luciferase (VvD14c-cLUC). For co-expression of VvMAX2 and VvD14c, we fused VvMAX2 with N-terminal luciferase (VvMAX2-nLUC) and VvD14c with C-terminal luciferase (VvD14c-cLUC*)* in *Nicotiana benthamiana* leaves, which resulted in strong luciferase activity. Conversely, no luciferase activity was observed in the combinations of VvMAX2-nLUC and cLUC, VvD14c-cLUC and nLUC, and cLUC and nLUC. These findings provide compelling evidence for the specific interaction between VvMAX2 and VvD14c (Fig. 4C).

### Roles of *VvMAX2* in regulating grapevine root architecture

**Fig. 5:**
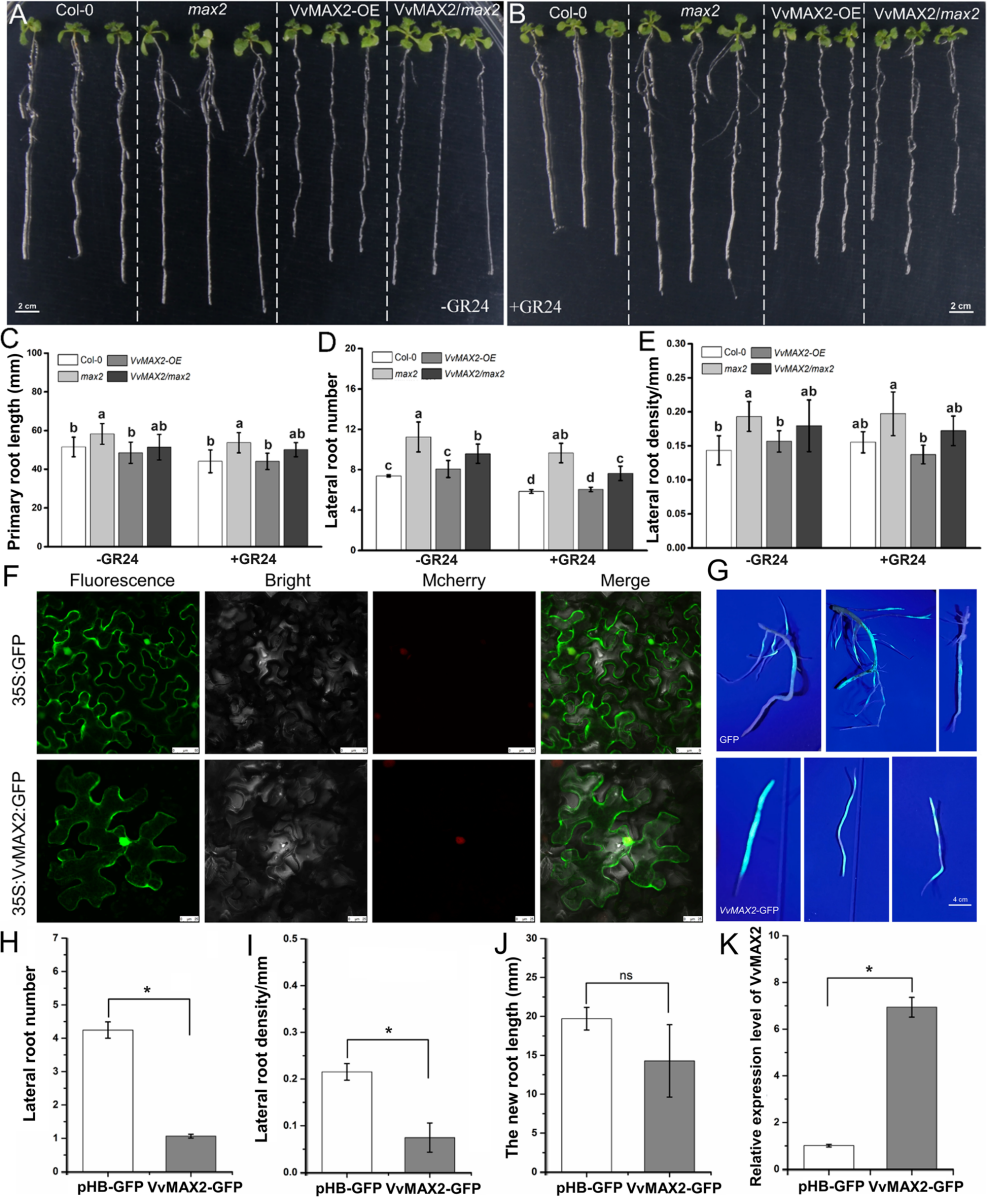
*VvMAX2* negatively regulates lateral root number and density in *Arabidopsis* roots. **A** Phenotypes of Col-0, *max2*, *VvMAX2-OE*, and *VvMAX2/max2* roots grown on 1/2 MS medium. Scale bar, 2 cm. **B** Phenotypes of Col-0, *max2*, *VvMAX2-OE*, and *VvMAX2/max2* roots grown on 1/2 MS medium containing 5 µM GR24. Scale bar, 2 cm. **C–E** Changes in the primary root length (**C**), lateral root number (**D**), and density (**E**) under GR24 or control medium in Col-0, *max2*, *VvMAX2-OE*, and *VvMAX2/max2*. Data in (**C–E**) are the mean ± SD of values from at least 50 biological replicates. **F** Subcellular localization of the VvMAX2 protein. The 35S: GFP was used as the positive control. Green fluorescence represents the GFP fusion protein signal. Red fluorescence represents the nucleus marker p2300-mCherry. Yellow indicates merged signals. **G** Phenotypes of GFP and *VvMAX2-OE* hairy roots after 80 days of *Agrobacterium rhizogenes* infection. Scale bar, 4 cm. **H–J** Changes in the lateral root number (**H**), density (**I**), and new root length (**J**) in GFP and *VvMAX2-OE* hairy roots. Data in (**H–J**) are the mean ± SD of values from at least 30 biological replicates. **K** Expression of *VvMAX2* was detected using RT-qPCR (reverse transcription-quantitative polymerase chain reaction) in GFP and *VvMAX2*-OE hairy roots. Data in (**K**) are the mean ± SD of values from three biological replicates. Significant differences were assessed using Student’s *t*-test (*p < 0.05)

*VvMAX2* exhibited high gene expression levels across different tissues and developmental stages in grapevines (Additional File 1: Fig. S4). In *Arabidopsis*, the *max2* mutant showed a greater number and density of LRs as well as longer PRs than the Col-0 seedlings without GR24 treatment (Fig. 5A–E). Interestingly, the LR density and PR length of *VvMAX2*/*max2* plants were similar to those of Col-0 seedlings, suggesting that VvMAX2 proteins act as negative regulators of LR density and PR length (Fig. 5A–E). Notably, significant differences persisted in PR length and LR number between the Col-0 and *max2* plants after treatment with 5 µM GR24, indicating that exogenous GR24 did not restore the root phenotype in the *max2 Arabidopsis* mutant (Fig. 5A–E). These results indicate that *VvMAX2* plays an essential role in grapevine root architecture. Additionally, the *max2 Arabidopsis* mutant exhibited more branches, longer petioles and wider leaves, although the leaves were shorter than those of Col-0 plants. In constrast, petiole length, leaf length, and width were not significantly different between the *VvMAX2*/*max2* and Col-0 plants (Additional File 1: Fig. S5). To further explore the biological function of VvMAX2, its subcellular localization was investigated using confocal microscopy. The 35S:VvMAX2:GFP fusion protein fluorescence signal was detected in both the cytoplasm and nucleus (Fig. 5F), consistent with predictions made using the WoLF PSORT prediction online tool (https://www.genscript.com/wolf-psort.html). To verify the functionality of VvMAX2 in grapevine, we performed  an  agroinfiltration assay on grapevine cuttings. The VvMAX2-overexpressing (OE) plants exhibited reduced LR number and density when compared with the control plants (Fig. 5G–J). Moreover, the expression of *VvMAX2* was significantly higher in *VvMAX2*-OE hairy roots than in root expressing the GFP empty vector roots (Fig. 5K).

### VvMAX2 interacts with VvLOB and VvLBD19 *in vivo* and *in vitro*

**Fig. 6:**
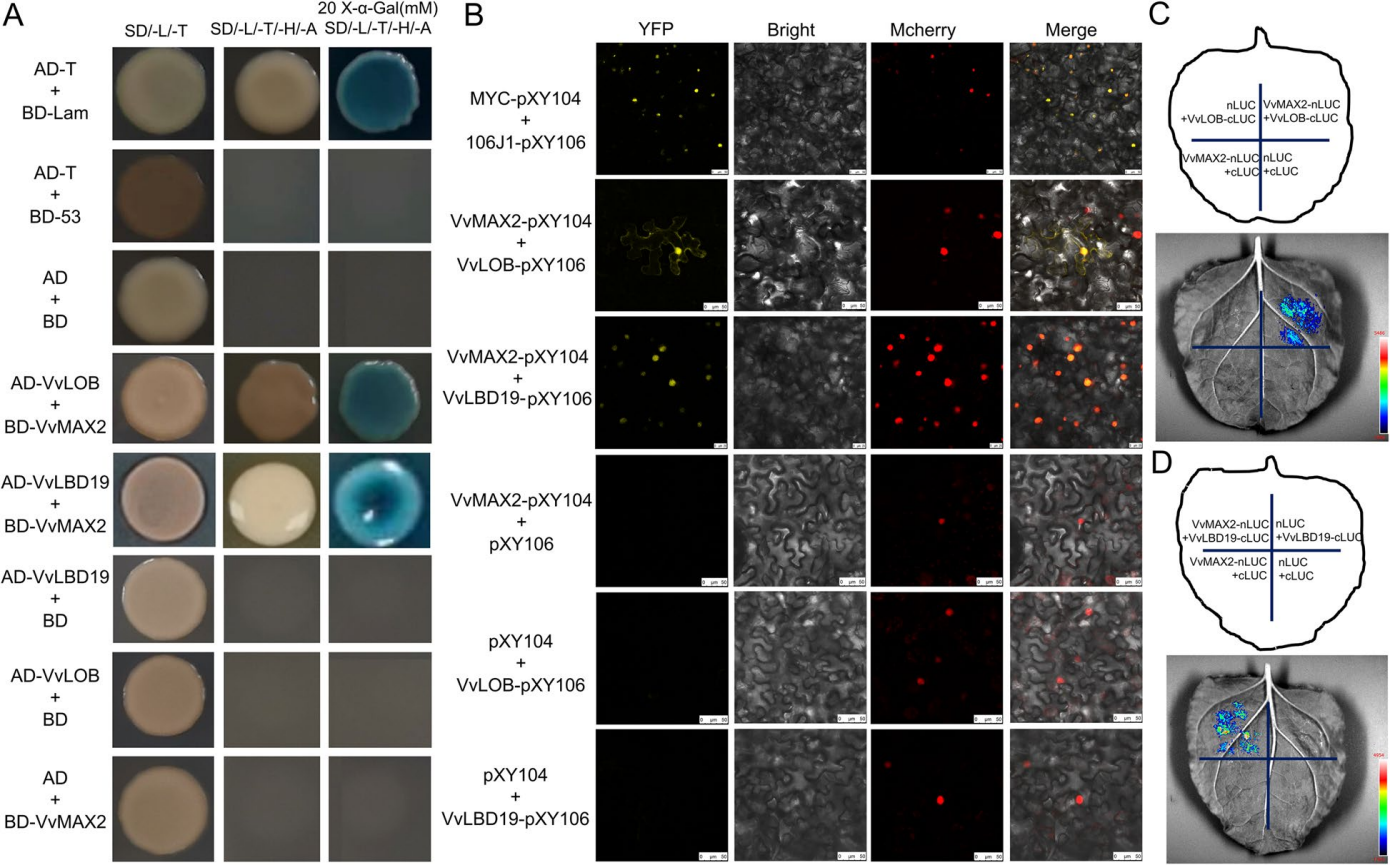
VvMAX2 physically interacts with VvLOB and VvLBD19.** A** Yeast two-hybrid assay corroborating the interaction between VvMAX2 and both VvLOB and VvLBD19. The yeast cells were cultured on SD/-Leu/-Trp medium for 2 days or on SD-Leu/-Trp/-Ade/-His medium with or without 20 mM X-α-gal for 5 days. **B** Bimolecular fluorescent complementation assay demonstrating the interaction of VvMAX2 with VvLOB and VvLBD19. Full-length VvMAX2 was fused with the split N-terminal (nYFP) fragment, while VvLOB and VvLBD19 were fused with the split C-terminal (cYFP) fragments of YFP. Bars: = 25 μm. p2300-mCherry served as the nuclear marker. **C–D** Split luciferase complementation assay (SLCA) illustrating the interaction between VvMAX2 and both VvLOB and VvLBD19. VvMAX2-nLUC and VvLOB-cLUC or VvMAX2-nLUC and VvLBD19-cLUC constructs were used to co-transform into tobacco leaves, and the LUC signal was detected after 48 h. Representative images are presented in the top panel, and luciferase activity is displayed in the bottom panel

To elucidate the regulatory mechanism of VvMAX2 in the SL signaling pathway affecting grapevine root architecture, we performed a Y2H assay to identify its interacting proteins. *VvMAX2 *were fused with the pGBKT7 vector, while *VvLBDs* (*VvLBD4*, *6*, *16*, *18*, *19*, *20*, *21*, and *27*) and *VvLOB* were fused with the pGADT7 vector. The Y2H assay showed that VvLOB and VvLBD19 interacted with VvMAX2 in vitro (Fig. 6A; Additional File 1: Fig. S6.). To confirm these interactions in vivo, we performed a BiFC assay, which demonstrated a strong yellow fluorescent protein (YFP) signal in tobacco cells co-expressing VvMAX2-cYFP and nYFP-VvLOB or nYFP-VvLBD19 (Fig. 6B). Conversely, no YFP signal was detected in the control combinations, confirming the specificity of the interaction. To further validate the interaction between VvMAX2, VvLOB/VvLBD19, we performed an split luciferase complementation assay (SLCA) This assay revealed robust luciferase activity in *N. benthamiana* leaves co-expressing VvMAX2-nLUC with VvLOB-cLUC or VvMAX2-nLUC and VvLBD19-cLUC (Fig. 6C–D). No luciferase activity was observed in the control combinations (Fig. 6C–D). Together, these results provide strong evidence that VvMAX2 interacts with VvLOB, and VvLBD19 both in vivo and in vitro.

### Roles of *VvLOB* and *VvLBD19* in regulating grapevine root architecture

**Fig. 7:**
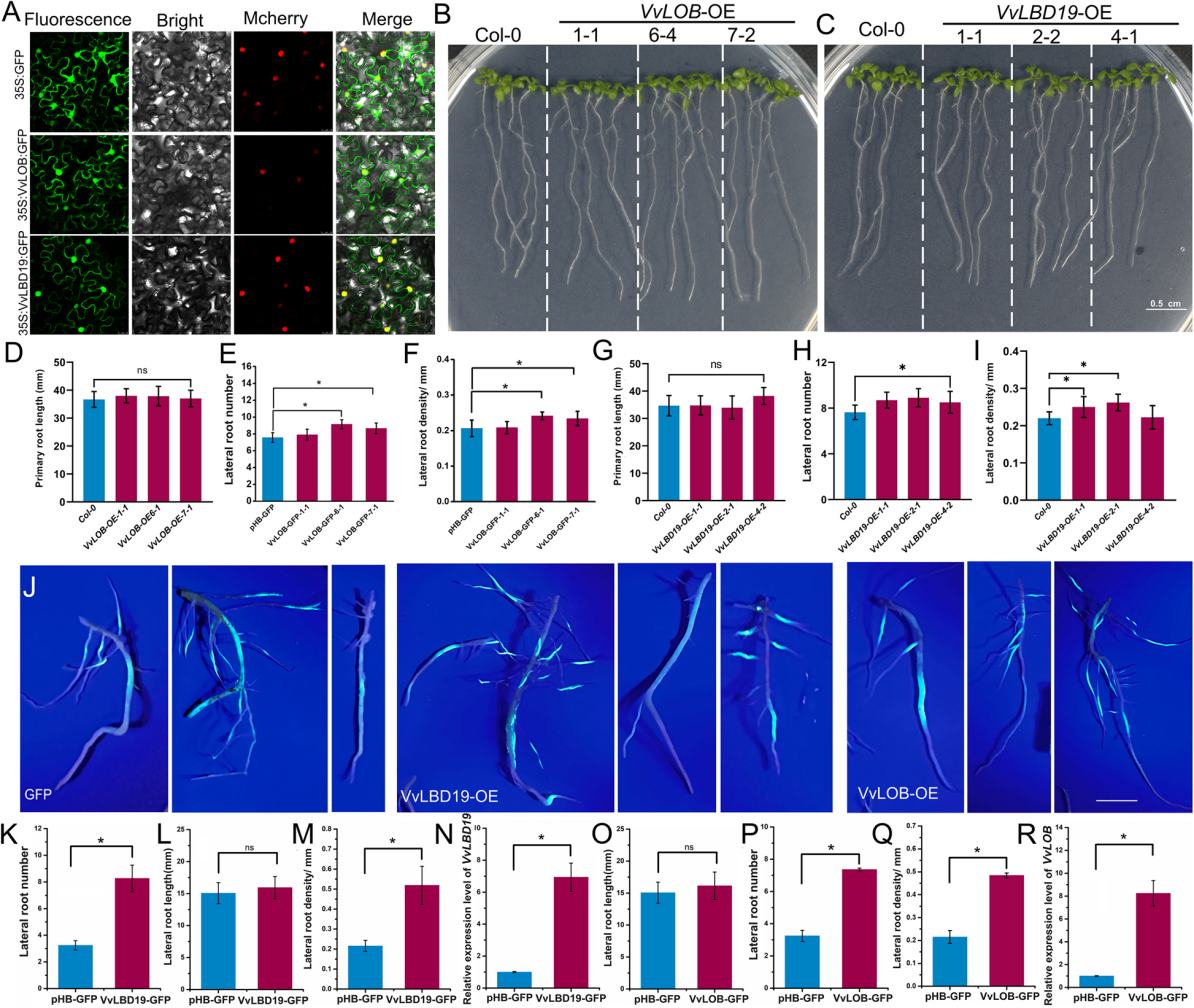
*VvLOB* and *VvLBD19* positively regulate grapevine LR density.** A** Subcellular localization of the *VvLOB* and *VvLBD19* proteins in tobacco leaf epidermal cells. The 35S: GFP was used as the positive control. Green fluorescence represents the GFP fusion protein signal. Red fluorescence represents the nuclear marker p2300-mCherry. Bars: 25 μm. Yellow indicates merged signals. **B-C** Phenotypes of Col-0, *VvLOB-OE* (**B**), *VvLBD19-OE* (**C**) roots grown on 1/2 MS medium. Scale bar, 0.5 cm. **D-I** Changes in the primary root length (**D**), lateral root number (**E**), and density (**F**) in Col-0, *VvLOB-OE*1, 6, 7 lines. Changes in the primary root length (**G**), lateral root number (**H**), and density (**I**) in Col-0, *VvLBD19-OE*1, 2, 4 lines. Data in (**D-I**) represent the mean ± SD of values from 50 biological replicates. Statistically significant differences were assessed using Student’s *t*-test (*p* < 0.05). **J** Phenotypes of GFP, *VvLBD19-OE*, and *VvLOB-OE* hairy roots 80 days post-*Agrobacterium rhizogenes* infection. Scale bar, 4 cm. **K-M **Changes in lateral root number (**K**), new root length (**L**), and lateral root density (**M**) in GFP and *VvLBD19-OE* hairy roots. **N** Expression of *VvLBD19* was detected using reverse transcription quantitative PCR (RT-qPCR) in GFP and *VvLBD19-OE* hairy roots. **O-Q** Changes in lateral root number (**O**), new root length (**P**), and lateral root density (**Q**) in GFP and *VvLOB-OE* hairy roots. **R** Expression of *VvLOB-OE* was detected by RT-qPCR in GFP and *VvLOB-OE* hairy roots. Data in (**K-M; O-Q**) represent the mean ± SD of values from at least 30 biological replicates. Data in (**N; R**) represent the mean ± SD of values from three biological replicates. Statistically significant differences were assessed using Student’s *t*-test (*p* < 0.05)

**Fig. 8:**
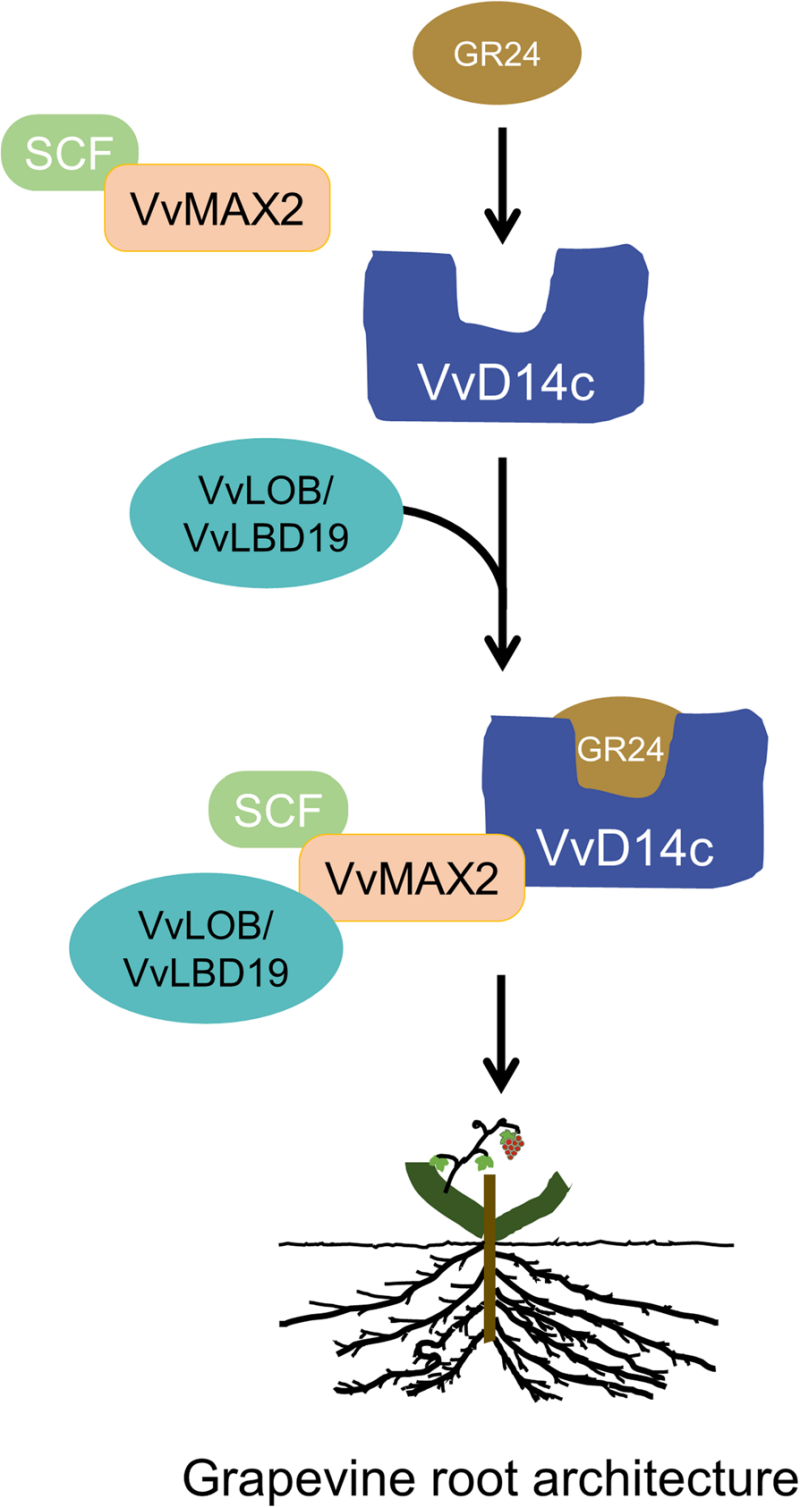
Model of the signaling pathway of Strigolactones (SLs) in the modulation of root architecture of grapevine. When SLs bind to their receptor, VvD14c, they undergo conformational changes and become active forms. These activated receptors then recruit VvMAX2, an F-box protein, and form the S-phase kinase-associated protein 1 (SKP1)-Cullin 1-F-box protein (SCF)-MAX2/D3 complex. Furthermore, VvMAX2 physically interacts with VvLOB and VvLBD19, which play positive roles in regulating LR density in grapevine

To elucidate the functions of VvLOB and VvLBD19, we conducted an analysis of their subcellular localization. Confocal microscopy revealed that the fluorescence signal of the 35S:VvLOB:GFP and 35S:VvLBD19:GFP fusion proteins were localized to both the cytoplasm and nucleus (Fig. 7A). To further understand their roles, we overexpressed these genes in *Arabidopsis* (Fig. 7B-I; Additional File 1: Fig. S7-8.). Interestingly, the overexpression of both VvLOB and VvLBD19 resulted in similar phenotypes, with increased LR number and density compared with those in Col-0 plants. To validate these functions in grapevine, *VvLBD19*-OE, *VvLOB*-OE, and the GFP positive control were agroinfiltrated into grapevine cuttings. *VvLBD19* and *VvLOB* transgenic hairy roots exhibited significantly higher LR number and density than the control (Fig. 7J-R). Furthermore, the expression levels of *VvLOB* and *VvLBD19* were significantly elevated in *VvLOB*-OE and *VvLBD19*-OE hairy roots compared with those in the GFP empty vector control (Fig. 7N; R). These findings highlight the critical role of the *VvLBD* gene family, particularly VvLOB and VvLBD19, in regulating grapevine root architecture, especially through their interaction with VvMAX2 (Fig. 8).

## Discussion

### D14 is involved in plant growth and development

*GhD14A/D* is highly expressed in cotton flowers, stems, leaves, and fibers 20 days after anthesis but is weakly expressed in cotton roots (Zhu et al. 2021). Similarly, *DAD2*, the *D14* ortholog in petunia, exhibits high expression in the axillary buds and leaves but low expression in the roots (Hamiaux et al. 2012). Our results align with these findings, revealing that *VvD14d* and *VvD14e* expression was lower in young grapevine roots compared to other tissues. In contrast, *MdD14* expression in apple is significantly higher in roots than in shoots. Interestingly, *VvD14a, VvD14b, and VvD14c* expression was higher in grapevine roots than in young stems (Additional File 1: Fig. S1.), suggesting that different *VvD14* homologs in grapevines may have tissue-specific roles.

The D14 protein regulates various plant growth processes, including hypocotyl elongation (Waters et al. 2012), mesocotyl elongation (Kameoka and Kyozuka 2015), cotyledon expansion (Waters et al. 2012), tillering (Umehara et al. 2008), branch angle (Liang et al. 2016), leaf senescence (Woo et al. 2001), stomatal closure (Bu et al. 2014), and anthocyanin/flavonoid production (Brewer et al. 2009). The double mutant of *N. tabacum Nbd14a*,* b* exhibited phenotypes consistent with the loss of SL perception in other plants, such as increased axillary bud outgrowth, reduced height, shortened petioles, and smaller leaves (White et al. 2022). Furthermore, D14 in tomato has been shown to regulate root development (Li et al. 2022a). Phylogenetic analysis revealed that *NtDAD2* from *N. tabacum* clustered with *SlD14* from *S. lycopersicum*, both of which are closely related to *VvD14c*, suggesting their comparable functions (Additional File 1: Fig. S2.). In the present study, GR24 treatment enhanced the elongation and diameter of ARs while inhibiting the elongation and density of LRs in grapevines (Fig. 2). This was accompanied by the upregulation of *VvD14c*. Additionally, molecular docking analysis further indicated that VvD14 serves as a potential receptor for GR24, with VvD14c protein showing a strong bounding affinity of 5.65 × 10^−9^M for GR24, aligning with previous studies and highlighted the conserved function of D14 is conserved among different species (Zhao et al. 2015). Furthermore, *VvD14c* was found to negatively regulate LR number and density while positively modulating plant length, and leaf length and width in *Arabidopsis* (Fig. 3; Additional File 1: Fig. S3.).

### SLs regulate root architecture in grapevines

SL biosynthesis primarily occurs in plant roots (Wheeldon and Bennett 2021). The regulatory effects of SLs on root architecture depend on growth conditions, such as sugar availability, inorganic phosphate, and nitrogen concentrations, as well as the plant species (Sun et al. 2022). These regulatory mechanisms are complex and influenced by interactions with auxin, cytokinin, and ethylene, as well as their levels, transport, and signaling pathways. The interactions between SLs and these plant hormones vary depending on the species (Xi et al. 2013). Compared to *Arabidopsis* wild-type plants, SL-deficient (*max1*, *max3*, *max4*, and *d14*) mutants exhibited longer LRs and increased LR quantity under normal growth conditions. Similarly, the *d14 Arabidopsis* mutant demonstrated increased LR number, density, and PR length (Fig. 3). Overexpression of *VvD14c* in grapevines reversed the phenotype of the *d14 Arabidopsis* mutant, revealing that *VvD14c* serves as a negative regulator of grapevine root architecture.

GmMAX2 interacts with GmD14 to form functionally active D14/KAI-SCF^MAX2^ complexes in soybeans (Ahmad et al. 2020). The D14-SCF^MAX2^ complex targets the class I Clp-ATPase repressor D53 in rice and *Arabidopsis* SMXL6, 7, and 8. Once targeted, these proteins are degraded via the ubiquitin-proteasome pathway (Wang et al. 2020a; Zhou et al. 2015). The degradation of SMXL6, 7, and 8 removes inhibitory effects on downstream genes, including *BRC1* and *PAP1*. With the full activation of stress responses, these genes also regulate plant growth and development (Wang et al. 2015, 2020a). Similarly, our findings demonstrate that VvD14c interacts with VvMAX2 in a GR24-dependent manner (Fig. 4). The expression of *MAX2* is not restricted to axillary shoots or branching points, as it is expressed throughout the plant, with peak expression in the developing vasculature (Stirnberg et al. 2002). This broad expression pattern, along with the mutant phenotype, suggests that MAX2 plays diverse roles in various plant tissues. The *max2* mutant phenotype is characterized by increased shoot branching and altered root elongation (Stirnberg et al. 2002; Ruyter-Spira et al. 2011). Grapevine *VvMAX2* rescued the phenotypic defects of *max2 Arabidopsis* mutants, suggesting a potential similarity in the functions of *VvMAX2* and *AtMAX2* (Fig. 5; Additional File 1: Fig. S5.).

### VvMAX2 and VvLOB/VvLBD19 interaction is likely involved in SL-mediated grapevine root architecture

The LBD gene family encodes plant-specific transcription factors that are involved in the development of lateral organs in higher plants (Xu et al. 2016). In *Arabidopsis*, LR formation depends on auxin response factors, such as *AtARF7* and *AtARF19*, which interact with *AtMYB77* (Shin et al. 2007). LR formation is hindered when these factors are disrupted, as observed in the *arf7arf19* double-knockout mutant. However, the defects in LR formation witnessed this double mutant were partially rescued by overexpression of either *AtLBD16* (*AtASL18*) or *AtLBD29* (*AtASL16*) (Okushima et al. 2007). *AtARF7* and *AtARF19* directly stimulate the expression of *AtLBD29* and *AtLBD16* (Okushima et al. 2007), thereby exerting a direct or indirect influence on the transcription of *AtLBD18 (AtASL20)* (Okushima et al. 2005; Lee et al. 2009). Previous studies have suggested that some members of the LOB gene family, namely, *LBD36*, *LOB*, and *LBD12*, are involved in the development of various lateral organs from the meristem in *Arabidopsis* plants (Shuai, 2002). *LBD19* transcripts were detected in the shoots, roots, and floral tissues but not in stems or leaves (Shuai, 2002). Meanwhile, AtLBD19 is a transcription factor involved in the regulation of root development in plants (Liu et al. 2019). Our study showed that *VvLOB* and *VvLBD19* played positive regulatory roles in the occurrence of grapevine LRs. Additionally, LBD regulates root development in conjunction with other plant hormones. For example, in *Arabidopsis*, the LBD genes responsive to cytokinins include *AtASL9 (AtLBD3)*, which is predominantly expressed at the base of lateral shoot organs, whereas its closely related counterpart, *AtASL6 (AtLOB)*, exhibits a comparatively lower level of expression in the roots. *AtASL9* (*AtLBD3*) is directly activated by type-B ARR transcription factors, making it a primary target for cytokinin-mediated phosphorylation signal transduction. Additionally, *AtAS2 (AtLBD6)* increases gibberellin synthesis by repressing *KNOX* genes in *Arabidopsis* (Ikezaki et al. 2010). Moreover, the class II *LBD* gene *AtASL37 (AtLBD40)* is downregulated by gibberellin and upregulated by DELLA, indicating its involvement in hormonal signaling networks (Zentella et al. 2007). *LBD3* gene overexpression significantly enhanced *MAX4* expression and reduced *MAX2* expression, suggesting that *LBD3* inhibits apical dominance through the MAX pathway. It has been hypothesized that *LBD3* functions by regulating auxin polar transport. However, few studies have verified an association between the LBD family and the SL hormone pathway through detection of protein interactions. Based on our results and previous research, we propose a working model in which, in grapevine, the D14-SCF^MAX2^ complex may tag and degrade transcription factors, such as VvLOB/VvLBD19, via the polyubiquitin/proteasome pathway, relieving the promoting effect of VvLOB/VvLBD19 on LRs. The molecular mechanism underlying the of SL-mediated development of grapevine root architecture in which VvLBDs are involved requires further exploration.

## Materials and methods

### Plant material and SL treatment

The grapevine cultivar ‘*Thomson seedless*’ was cultivated in vitro at 23 ℃ under a 16-hour light/8-hour dark photoperiod at the Shanghai Jiao Tong University, Shanghai, China (31°11’ N, 121°29’ W). The plantlets were propagated on Murashige and Skoog (MS) medium supplemented with 3% (w/v) sucrose, 0.5 mg/L indole-3-butyric acid, and 0.8% (w/v) agar. Prior to sterilization, the pH of the medium was adjusted to 5.8–6.0. To initiate AR formation before SL treatment, approximately 4 cm long shoot tips from the apex of the plants were cultured on MS medium containing 0.5 mg/L indole-3-butyric acid for 5 days. Subsequently, the stem tips were excised from the original growth medium, rinsed with sterile water 6–8 times, and transferred to a fresh MS medium supplemented with 10 µM GR24. The control stem tips were cultivated in MS medium devoid of GR24. Each treatment consisted of 50 grapevine tissue culture seedlings, with 10 biological replicates per treatment. The count, length, and diameter of ARs were measured on 4, 8, 16, 25, and 34 DAT. Furthermore, to investigate their morphological characteristics, LRs were randomly selected to assess their count, length, and density. Samples were collected at 4, 8, 16, 25, and 34 DAT, immediately flash-frozen in liquid nitrogen, and stored at -80 ℃ until further analysis.

The ripe berry, old leaves, young leaves, mature leaves, young stems, young roots, old roots, seeds, flower axis, and flowers of ‘*Summer Black*’ were collected to analyze *VvD14s* expression. The samples were subsequently flash-frozen in liquid nitrogen and stored at − 80 ℃ until subsequent RNA extraction.

## Quantitative real-time polymerase chain reaction (qRT-PCR)

Total RNA was extracted from grapevines using the CTAB method (Jiu et al. 2016). RNA concentration and quality were assessed using NanoDrop 2000 (Thermo Fisher Scientific Inc.) and agarose gel electrophoresis, respectively. To prevent genomic DNA contamination, the total RNA was digested with DNase I (TaKaRa Bio). Subsequently, cDNA libraries were established using 1.0 µg RNA samples (RR047Q Takara). The primers listed in Additional File 2: Table S1 were designed using Primer-BLAST. qRT**-**PCR was performed following a previously described method (Jiu et al. 2023).

### Phylogenetic tree and sequence analysis

The TAIR database (https://www.arabidopsis.org/) was utilized to identify *A. thaliana AtD14*, and grapevine homologs (*VvD14* genes) were identified using BLAST (https://blast.ncbi.nlm.nih.gov/Blast.cgi). *VvD14c* was cloned from ‘*Muscat Hamburg*’ grapevine root tissue, with primers listed in Additional File 2: Table S2. The sequences of VvD14 proteins are provided in Additional File 2 Table S3. Sequence alignment of *VvD14c* in grapevine, *AtD14* in *Arabidopsis*, *NtDAD2* in *N. tabacum*, *SlD14* in *S. lycopersicum*, *OsD14* in *Oryza sativa*, and *CsD14* in *Citrus sinensis* was conducted using EditSeq (DNAstar, Inc. package). MEGA v.7.0 (https://www.megasoftware.net/MEGA) was employed to construct and analyze the phylogenetic tree. The nucleic acid and protein sequences of the other six species were obtained from NCBI BLAST (https://blast.ncbi.nlm.nih.gov/Blast.cgi) based on the amplified *D14* DNA sequences, facilitating evolutionary phylogenetic comparisons (Liu et al. 2017). The Swiss model (https://swissmodel.expasy.org/) was used for the prediction of protein tertiary structure.

### Subcellular localization

The coding sequences (CDSs) of *VvD14c*, *VvMAX2*, *VvLOB*, *and VvLBD19*, excluding the termination codons, were amplified using the primers specified in Additional File 2: Table S4. The amplified CDSs were cloned into the binary vector pHB, which contains two cauliflower mosaic virus 35 S promoters, a translation enhancer, and a GFP tag. This cloning procedure yielded fusion constructs designated as p35S-VvD14c-GFP, p35S-VvMAX2-GFP, p35S-VvLOB-GFP, and p35S-VvLBD19-GFP. Following sequence verification, both the fusion constructs and control vector (pHB) were introduced into the *Agrobacterium tumefaciens* strain GV3101. Subsequently, these constructs were employed to infiltrate the leaves of tobacco plants aged 3–5 weeks. At three days post-infiltration, coinciding with peak GFP fluorescence, the localization of the fluorescent proteins was visualized using a confocal laser scanning microscope (Leica TCS SP8 STED 3X, Wetzlar, Germany), following the manufacturer’s guidelines.

### Computational docking

The flexible-ligand sampling algorithm (Mukherjee et al. 2010), integrated within the DOCK 6.7 software platform (Allen et al. 2015), facilitated the docking procedure of GR24 and its SL analog stereoisomers into the D14 ligand-binding pocket. During this process, the alignment of the D-Ring to the reference crystal structure dictated preferential ligand positioning. Subsequently, a comprehensive evaluation incorporated both the combined grid Van-der-Waals and electrostatic (vdw + es) scores and internal energy assessments to identify energetically advantageous docking conformations for each ligand. Before the docking stage, the all-atom AMBER ff14SB3 parameter set (Maier et al. 2015) assigned partial charges to the receptor, while semi-empirical AM1-BCC charges, tailored for each SL analog, ensured accurate electrostatic interactions during the simulation.

### VvD14c receptor binding assay with GR24 hormone

The *VvD14c* gene was constructed using the pET-28b vector and cloned into Rosetta (DE3) *Escherichia coli* for inducing expression at 16 °C and 180 rpm for 16–18 h with an IPTG concentration of 0.5 mM. For protein purification, refer to the His-tag purification resin (Beyotime, P2210) instructions for the method operation. The LMW multi-cycle kinetics/affinity method was performed using a Biacore 8 K instrument at the Instrumental Analysis Center, Shanghai Jiao Tong University, with a chip type of CM5, a data collection frequency of 10 Hz, and a running buffer of PBST + 5% DMSO.

### Stable plant transformation

The full-length CDSs of *VvD14c*, *VvMAX2*, *VvLOB*, *and VvLBD19* were cloned into the binary vector pHB-GFP and used to transform *Agrobacterium* strain GV3101. Transgenic *Arabidopsis* plants were acquired using the floral dip method and stably transformed into *Arabidopsis* accession Col-0 (Clough and Bent 1998). The transgenic plants were grown in a plant growth chamber with a 16-hour light/8-hour dark photoperiod at 23 °C. Following screening on a selective medium and DNA detection, we selected transgenic plants demonstrating high expression levels for subsequent phenotypic analyses. *Agrobacterium rhizogenes* MSU440-mediated grape hairy root transformation was performed according to the protocol described by Gao et al. (2023).

### Yeast two-hybrid analysis

The full-length CDSs of *VvD14c* were cloned into the pGBKT7 vector, while *VvMAX2* was inserted into the pGADT7 vector. Sequencing confirmed the authenticity of all reconstructed vectors. Subsequently, the pGADT7 and pGBKT7 vectors were introduced into Y2Hgold cells. The screening of protein interactions was performed on a medium lacking tryptophan (Trp), histidine (His), leucine (Leu), and adenine (Ade) but supplemented with 20 mM X-α-Gal and 5 µM GR24. Additional File 2, Table S4 provides detailed information about the primers used for Y2H vector construction.

### Bimolecular fluorescence complementation

Using the method described by Li et al. (2022a), Agrobacterium colonies carrying a fusion construct of nYFP and cYFP were infiltrated into *N. benthamiana* leaves. YFP fluorescence was captured using a Leica TCS SP8 STED 3X confocal microscope with an excitation wavelength of 514 nm and an emission wavelength of 520 nm. Additional File 2: Table S4 provides details of the primers used to construct the pXY104 and pXY106 vectors.

### Split luciferase complementation assay

To improve clarity and minimize redundancy, the full-length CDSs of the genes were cloned into N-terminal luciferase (nLUC) or C-terminal luciferase (cLUC) vectors. The resulting constructs were then transformed into the *A. tumefaciens* strain GV3101 and infiltrated into *N. benthamiana* leaves. After 2 days, luciferase reporter assays were conducted using commercially available luciferase reporter reaction reagents (Yeason).

### Statistical analysis

SAS (Statistical Analysis System) v.9.3 (SAS Institute Inc., Cary, NC, USA) was used for all data analyses. Results are presented as means ± standard deviation. Statistical significance was set at *p* < 0.05.

## Supplementary Information

Additional File 1. Fig. S1. Identification of the tissue-specific and fruit development stage-specific expression of VvD14a-e. A–E Tissue-specific expression of VvD14a (A), VvD14b (B), VvD14c (C), VvD14d (D), and VvD14e (E). F–J The expression levels of VvD14a (F), VvD14b (G), VvD14c (H), VvD14d (I), and VvD14e (J) at 7, 28, 49, 57, 63, and 94 days after anthesis. K Semi-quantitative expression of VvD14a-e at different stages of fruit development. L Semi-quantitative expression of VvD14a-e in various grapevine tissues. Data represent the mean values from three replicates, and error bars indicate standard errors. Mean values with the same letters are not significantly different (Tukey's test, *p* < 0.05). Ripe berry (RB), old leaves (OL), young leaves (YL), mature leaves (ML), young stems (YS), young roots (YR), old roots (OR), seeds (SE), flower axis (FA), and flowers (Fl). Fig. S2. Evolutionary analysis of VvD14c. Fig. S3. VvD14c positively regulates main stem length, leaf length, leaf width, and petiole length in *Arabidopsis*. Fig. S4. RNA-seq results (GEO Accession: GSE36128) demonstrate the expression profiles of strigolactone synthesis and signal transduction pathway genes in grapevine tissues at different developmental stages. Fig. S5. VvMAX2 regulates main stem length, leaf length, leaf width, and petiole length in *Arabidopsis*. Fig. S6. Verification of interactions between VvMAX2 and other VvLBD proteins related to grapevine root growth and development. Fig. S7. Aboveground phenotypes of Col-0 and VvLOB-OE plants. Fig. S8. Aboveground phenotypes of Col-0 and VvLBD19-OE plants.

Additional File 2. Table S1. Primer sequences used for quantitative reverse transcription polymerase chain reaction (qRT-PCR). Table S2. Primer sequences for gene cloning. Table S3. Protein sequences used in this study. Table S4. Primer sequences used for vector construction

## Data Availability

The data will be available from the corresponding author upon reasonable request.

## Abbreviations

AR: Adventitious roots
ASL: ASYMMETRIC LEAVES2-LIKE
BiFC: Bimolecular fluorescence complementation
CDS: Coding sequence
cLUC: C-terminal luciferase
DAT: Days after treatment
LBD: LOB DOMAIN
LOB: Lateral organ boundary
LR: Lateral root
MAX2: MORE AXILLARY GROWTH 2
MS: Murashige and Skoog
NCBI: National Center for Biotechnology Information
nLUC: N-terminal luciferase
PAP1: PRODUCTION OF ANTHOCYANIN PIGMENT 1
PR: Primary root
qRT-PCR: Quantitative Real-Time Polymerase Chain Reaction
SKP1: S-phase kinase-associated protein 1
SLCA: Split-luciferase complementation assay
SMXL: SUPPRESSOR OF MAX2-LIKE
YFP: Yellow fluorescent protein
