# Supplementary figures and images for: VvD14c-VvMAX2-VvLOB/VvLBD19 module is involved in the strigolactone-mediated regulation of grapevine root architecture
Source: Mol Hortic. 2024 Oct 25;4:40. doi: 10.1186/s43897-024-00117-z (PMC11515387; doi:10.1186/s43897-024-00117-z)

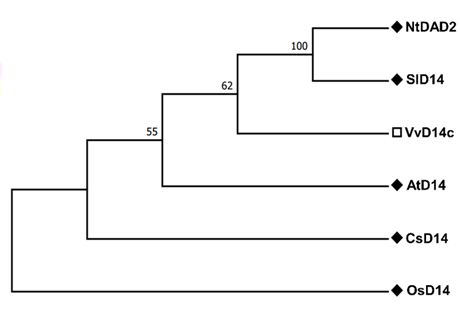

Supplement: Supplementary file 1 — Additional File 1. Fig. S1. Identification of the tissue-specific and fruit development stage-specific expression of VvD14a-e. A–E Tissue-specific expression of VvD14a (A), VvD14b (B), VvD14c (C), VvD14d (D), and VvD14e (E). F–J The expression levels of VvD14a (F), VvD14b (G), VvD14c (H), VvD14d (I), and VvD14e (J) at 7, 28, 49, 57, 63, and 94 days after anthesis. K Semi-quantitative expression of VvD14a-e at different stages of fruit development. L Semi-quantitative expression of VvD14a-e in various grapevine tissues. Data represent the mean values from three replicates, and error bars indicate standard errors. Mean values with the same letters are not significantly different (Tukey's test, p < 0.05). Ripe berry (RB), old leaves (OL), young leaves (YL), mature leaves (ML), young stems (YS), young roots (YR), old roots (OR), seeds (SE), flower axis (FA), and flowers (Fl). Fig. S2. Evolutionary analysis of VvD14c. Fig. S3. VvD14c positively regulates main stem length, leaf length, leaf width, and petiole length in Arabidopsis. Fig. S4. RNA-seq results (GEO Accession: GSE36128) demonstrate the expression profiles of strigolactone synthesis and signal transduction pathway genes in grapevine tissues at different developmental stages. Fig. S5. VvMAX2 regulates main stem length, leaf length, leaf width, and petiole length in Arabidopsis. Fig. S6. Verification of interactions between VvMAX2 and other VvLBD proteins related to grapevine root growth and development. Fig. S7. Aboveground phenotypes of Col-0 and VvLOB-OE plants. Fig. S8. Aboveground phenotypes of Col-0 and VvLBD19-OE plants. [file 43897_2024_117_MOESM1_ESM.zip › Additional file 1 Fig. S2.tif]

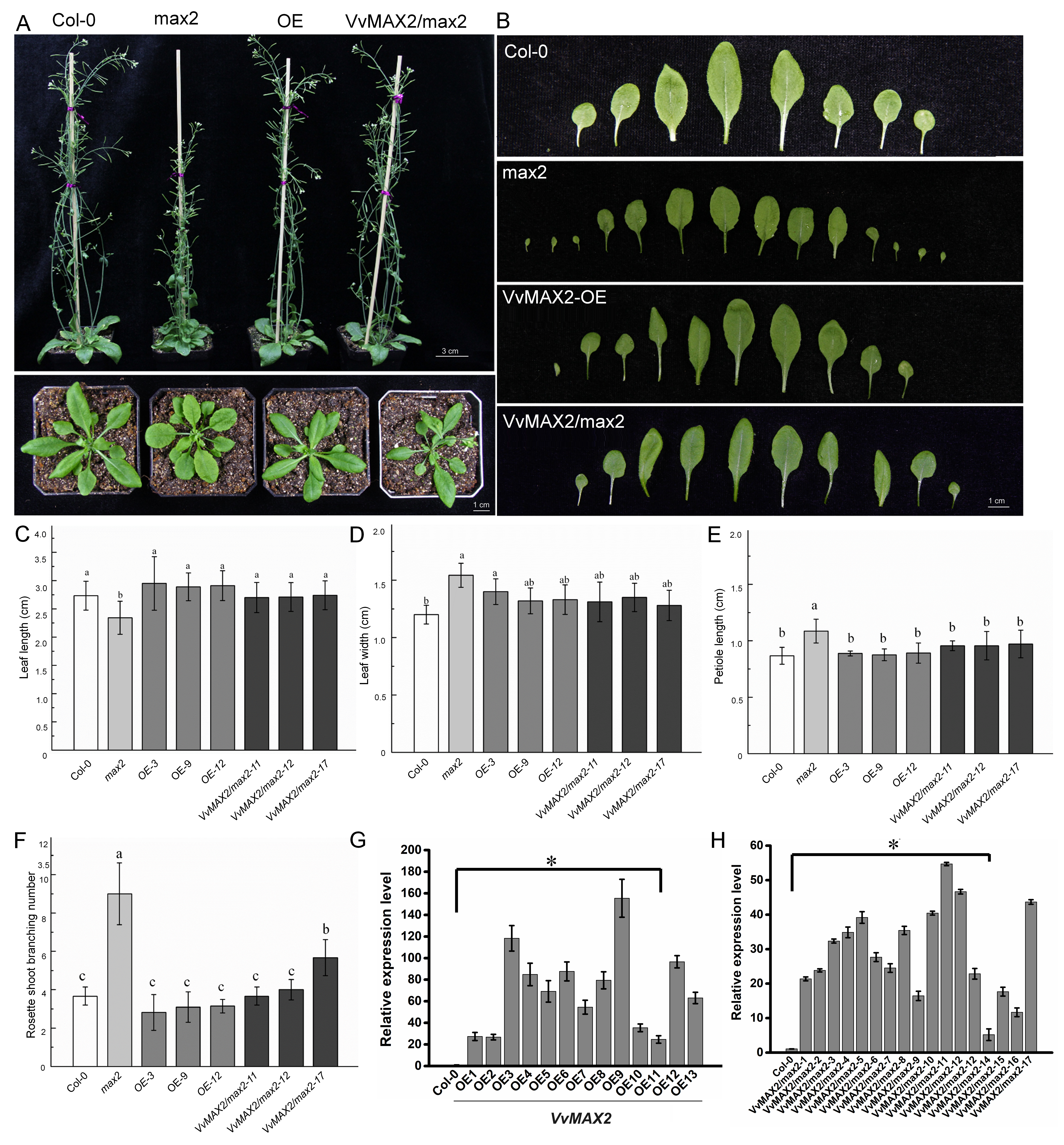

Supplement: Supplementary file 1 — Additional File 1. Fig. S1. Identification of the tissue-specific and fruit development stage-specific expression of VvD14a-e. A–E Tissue-specific expression of VvD14a (A), VvD14b (B), VvD14c (C), VvD14d (D), and VvD14e (E). F–J The expression levels of VvD14a (F), VvD14b (G), VvD14c (H), VvD14d (I), and VvD14e (J) at 7, 28, 49, 57, 63, and 94 days after anthesis. K Semi-quantitative expression of VvD14a-e at different stages of fruit development. L Semi-quantitative expression of VvD14a-e in various grapevine tissues. Data represent the mean values from three replicates, and error bars indicate standard errors. Mean values with the same letters are not significantly different (Tukey's test, p < 0.05). Ripe berry (RB), old leaves (OL), young leaves (YL), mature leaves (ML), young stems (YS), young roots (YR), old roots (OR), seeds (SE), flower axis (FA), and flowers (Fl). Fig. S2. Evolutionary analysis of VvD14c. Fig. S3. VvD14c positively regulates main stem length, leaf length, leaf width, and petiole length in Arabidopsis. Fig. S4. RNA-seq results (GEO Accession: GSE36128) demonstrate the expression profiles of strigolactone synthesis and signal transduction pathway genes in grapevine tissues at different developmental stages. Fig. S5. VvMAX2 regulates main stem length, leaf length, leaf width, and petiole length in Arabidopsis. Fig. S6. Verification of interactions between VvMAX2 and other VvLBD proteins related to grapevine root growth and development. Fig. S7. Aboveground phenotypes of Col-0 and VvLOB-OE plants. Fig. S8. Aboveground phenotypes of Col-0 and VvLBD19-OE plants. [file 43897_2024_117_MOESM1_ESM.zip › Additional file 1 Fig. S5.tif]

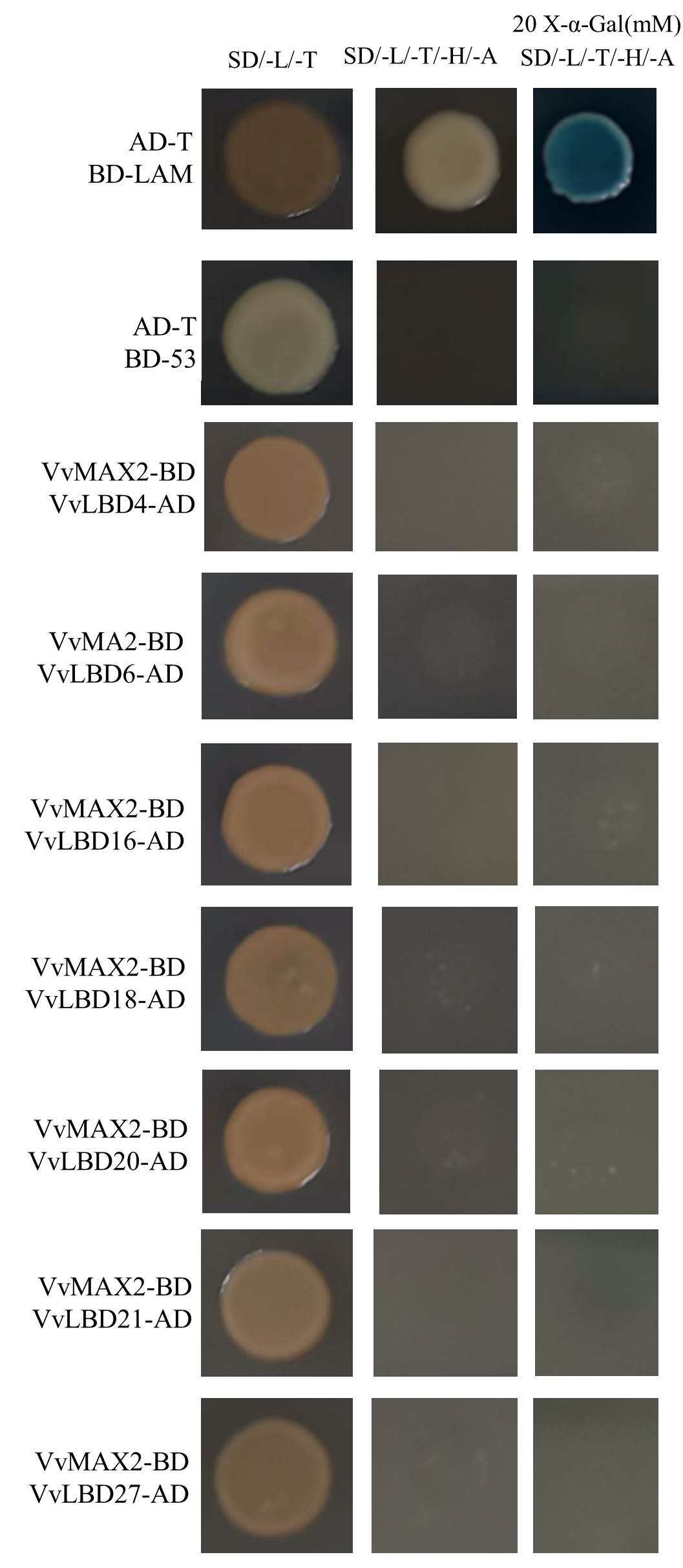

Supplement: Supplementary file 1 — Additional File 1. Fig. S1. Identification of the tissue-specific and fruit development stage-specific expression of VvD14a-e. A–E Tissue-specific expression of VvD14a (A), VvD14b (B), VvD14c (C), VvD14d (D), and VvD14e (E). F–J The expression levels of VvD14a (F), VvD14b (G), VvD14c (H), VvD14d (I), and VvD14e (J) at 7, 28, 49, 57, 63, and 94 days after anthesis. K Semi-quantitative expression of VvD14a-e at different stages of fruit development. L Semi-quantitative expression of VvD14a-e in various grapevine tissues. Data represent the mean values from three replicates, and error bars indicate standard errors. Mean values with the same letters are not significantly different (Tukey's test, p < 0.05). Ripe berry (RB), old leaves (OL), young leaves (YL), mature leaves (ML), young stems (YS), young roots (YR), old roots (OR), seeds (SE), flower axis (FA), and flowers (Fl). Fig. S2. Evolutionary analysis of VvD14c. Fig. S3. VvD14c positively regulates main stem length, leaf length, leaf width, and petiole length in Arabidopsis. Fig. S4. RNA-seq results (GEO Accession: GSE36128) demonstrate the expression profiles of strigolactone synthesis and signal transduction pathway genes in grapevine tissues at different developmental stages. Fig. S5. VvMAX2 regulates main stem length, leaf length, leaf width, and petiole length in Arabidopsis. Fig. S6. Verification of interactions between VvMAX2 and other VvLBD proteins related to grapevine root growth and development. Fig. S7. Aboveground phenotypes of Col-0 and VvLOB-OE plants. Fig. S8. Aboveground phenotypes of Col-0 and VvLBD19-OE plants. [file 43897_2024_117_MOESM1_ESM.zip › Additional file 1 Fig. S6.tif]

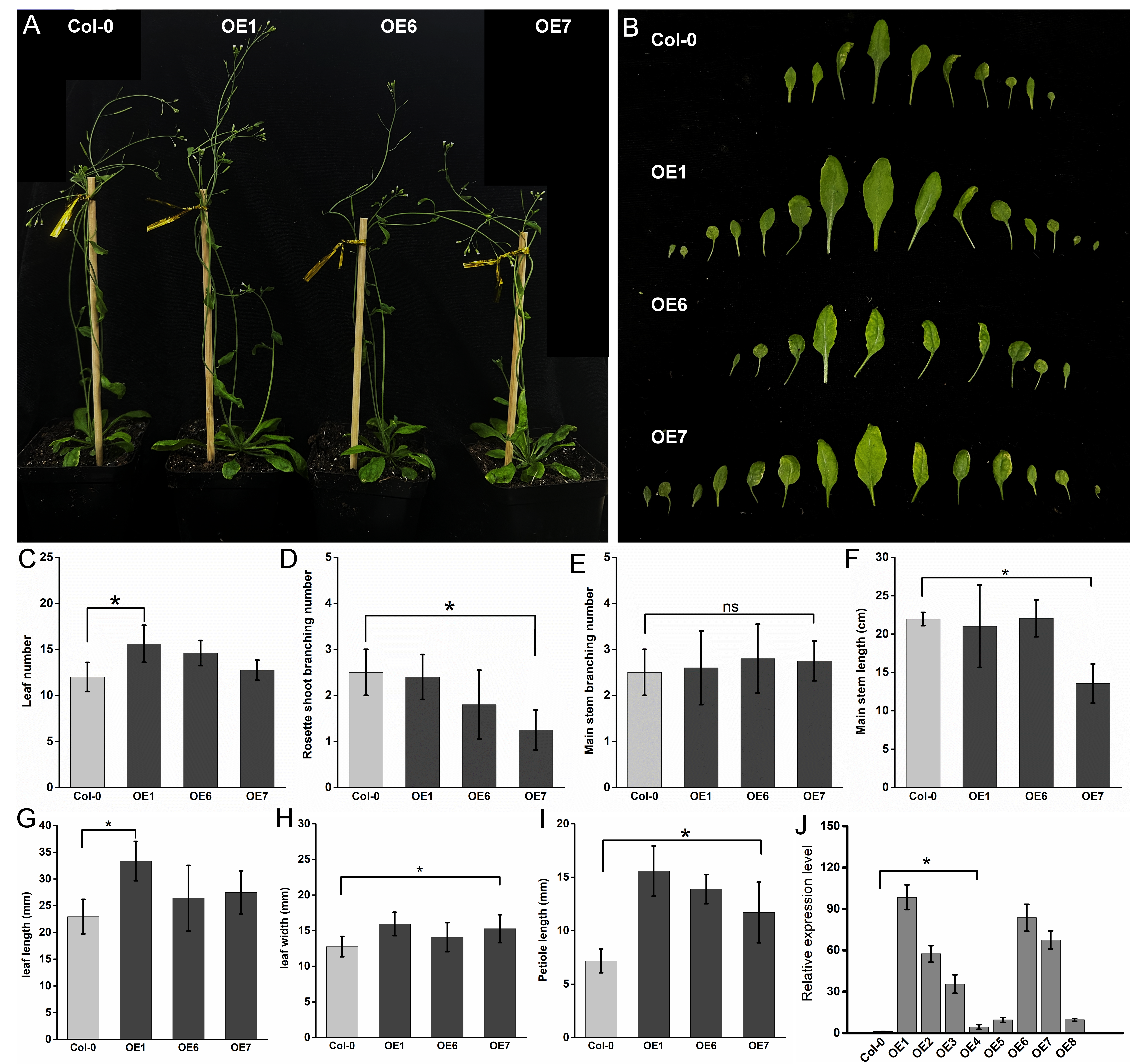

Supplement: Supplementary file 1 — Additional File 1. Fig. S1. Identification of the tissue-specific and fruit development stage-specific expression of VvD14a-e. A–E Tissue-specific expression of VvD14a (A), VvD14b (B), VvD14c (C), VvD14d (D), and VvD14e (E). F–J The expression levels of VvD14a (F), VvD14b (G), VvD14c (H), VvD14d (I), and VvD14e (J) at 7, 28, 49, 57, 63, and 94 days after anthesis. K Semi-quantitative expression of VvD14a-e at different stages of fruit development. L Semi-quantitative expression of VvD14a-e in various grapevine tissues. Data represent the mean values from three replicates, and error bars indicate standard errors. Mean values with the same letters are not significantly different (Tukey's test, p < 0.05). Ripe berry (RB), old leaves (OL), young leaves (YL), mature leaves (ML), young stems (YS), young roots (YR), old roots (OR), seeds (SE), flower axis (FA), and flowers (Fl). Fig. S2. Evolutionary analysis of VvD14c. Fig. S3. VvD14c positively regulates main stem length, leaf length, leaf width, and petiole length in Arabidopsis. Fig. S4. RNA-seq results (GEO Accession: GSE36128) demonstrate the expression profiles of strigolactone synthesis and signal transduction pathway genes in grapevine tissues at different developmental stages. Fig. S5. VvMAX2 regulates main stem length, leaf length, leaf width, and petiole length in Arabidopsis. Fig. S6. Verification of interactions between VvMAX2 and other VvLBD proteins related to grapevine root growth and development. Fig. S7. Aboveground phenotypes of Col-0 and VvLOB-OE plants. Fig. S8. Aboveground phenotypes of Col-0 and VvLBD19-OE plants. [file 43897_2024_117_MOESM1_ESM.zip › Additional file 1 Fig. S7.tif]
