# Supplementary material for: VvD14c-VvMAX2-VvLOB/VvLBD19 module is involved in the strigolactone-mediated regulation of grapevine root architecture
Source: Mol Hortic. 2024 Oct 25;4:40. doi: 10.1186/s43897-024-00117-z (PMC11515387; doi:10.1186/s43897-024-00117-z)
Supplement: Supplementary file 2 — Additional File 2. Table S1. Primer sequences used for quantitative reverse transcription polymerase chain reaction (qRT-PCR). Table S2. Primer sequences for gene cloning. Table S3. Protein sequences used in this study. Table S4. Primer sequences used for vector construction [file 43897_2024_117_MOESM2_ESM.zip › Additional file 2 Table S1.docx]

**Table S1 Sequence of primers used for** **qRT-PCR in this study.**

| **Gene Name** | **Forward primer sequences (5’→3’)** | **Reverse primer sequences (5’→3’)** |
| --- | --- | --- |
| *KyActin1* | GATTCTGGTGATGGTGTGAGT | GACAATTTCCCGTTCAGCAGT |
| *VvD14c* | GCGTTGATCGTTGCGCTTAT | CGTCGTAGTTGGCCTCCATT |
| *VvMAX2* | TCGGATTCAACGACTGCACA | AAGCGTCCAGATCAGTGGTG |
| *VvD14a* | AGGTGGCTCTCCAAGGTACT | ACCGTAGCATCGTCACCAAG |
| *VvD14b* | CGTCAGTTTTTGTCGGGCAC | GGGGTCGTAACCTTGTCGAG |
| *VvD14d* | CGTTGAGTTCAACTTGCGGG | GAATGTCACGTTCGTCGCAG |
| *VvD14e* | TGGGCTTCATACTCTGCTTCT | TGCTTGACGACTCCAGATGAG |
| *VvLOB* | CTGGATGCATCTTTGCACCG | TGACATCTCATTGCAGGCGT |
| *VvLBD19* | CACAAGGTGTTTGGTGCCAG | CTTTCAGGCAGTAGGGGTGG |
| *AtMAX2* | CCGGAGAACGATATGAGCACAGAG | TTGGTCCTCGAATCGGCTACAC |
| *AtActin* | AGTGGTCGTACAACCGGTATTGT | GATGGCATGAGGAAGAGAGAAAC |
| *AtSMXL6* | CGGGCTATTGAGACCAAAGA | GGACTCACCGACTGAAAACC |
