# Supplementary material for: VvD14c-VvMAX2-VvLOB/VvLBD19 module is involved in the strigolactone-mediated regulation of grapevine root architecture
Source: Mol Hortic. 2024 Oct 25;4:40. doi: 10.1186/s43897-024-00117-z (PMC11515387; doi:10.1186/s43897-024-00117-z)
Supplement: Supplementary file 2 — Additional File 2. Table S1. Primer sequences used for quantitative reverse transcription polymerase chain reaction (qRT-PCR). Table S2. Primer sequences for gene cloning. Table S3. Protein sequences used in this study. Table S4. Primer sequences used for vector construction [file 43897_2024_117_MOESM2_ESM.zip › Additional file 2 Table S2.docx]

**Table S2** **Sequence of primers used for gene clone in this study.**

| **Gene Name** | **Forward primer sequences (5’→3’)** | **Reverse primer sequences (5’→3’)** |
| --- | --- | --- |
| *VvMAX2* | ATGGCCGGAGCTGCTGCCG | TCAATCAAGTATCCTCCGC |
| *VvLOB* | ATGGCTTCATCCAGCTCTTACAACTC | TCACATACTGCCTCCCCCT |
| *VvLBD19* | ATGACTGGAAGCAAGGGAGA | TTAGTTAGAACTCGAATCCC |
| *VvD14c* | ATGGGTAACACCCTCTTGG | TCACCGTGAGAGGGCACGA |
