# Supplementary material for: VvD14c-VvMAX2-VvLOB/VvLBD19 module is involved in the strigolactone-mediated regulation of grapevine root architecture
Source: Mol Hortic. 2024 Oct 25;4:40. doi: 10.1186/s43897-024-00117-z (PMC11515387; doi:10.1186/s43897-024-00117-z)
Supplement: Supplementary file 2 — Additional File 2. Table S1. Primer sequences used for quantitative reverse transcription polymerase chain reaction (qRT-PCR). Table S2. Primer sequences for gene cloning. Table S3. Protein sequences used in this study. Table S4. Primer sequences used for vector construction [file 43897_2024_117_MOESM2_ESM.zip › Additional file 2 Table S3.docx]

**Table S3 Protein sequence used in this study.**

>VvMAX2

MAGAAAGGATTIHDLPDAILSSILASVTDTRARNAAALVCRKWLVLERGTRTSLTLRGNVVHNNLYMIPTCFRAVTHLDLSLLSPWGHSLISPSSDPMLLAHLLRHAFPMVTSLTVYARTPATLQLLAPQWPNLTHIKLVKWHQRSPSALGSDFDPILRHCTSLTSVDLSNFYYWTEDLPPALQAHPATAAALTRLDLMTLSFAEGFKSHEILAITAACPNLQQLLIACTFDPRYIGFVGDEAIVAIASNCPGLTVLHLADTASLSNGRGDPEEEGFSSEDAGISTTALSGLFSGLPLLQELVLDVCKNVRDSGATLEMLNSRCPKLRVLKLGHFHGLCLAIGSQLDGVALCQGLESLSIKNSADLTDMGLIAIARGCSKLAKFEIHGCKKVTWKGISTMACLRRSTLVEFKISCCKNLDAVSALRGLEPIRDRIQRLHIDCIWDRSEQFEDSEEAILAHSFDLNELEQPSIPSQDDDRFWDHEASIKKKKRKYTTDLDASYEQNNGNGICSKTWERLRCLSLWIGVGELLPPLAKAGLDDCPCLEEIQIKVEGDCRERSKPSQPFGLSSLMRYPRLSKMKLDCGDTIGYALTAPSGQTDLSTWERFYLNGIKNLTLNELDYWPPQDKDVNHRSLSLPSAGLLAECVTLRKLFIHGTAHEHFMTFLLAIPNLRDVQLREDYYPAPENDMSTEMRIDSCSRFEDALNRRRILD*

>VvD14a

MNEGERVPSLKSQLVLVPLSQSFKALHCQLVKLQIANSKMEIRSIRQATPYHRDLAVSKSLLRNMDMLSESGGGIIEALNATVHGNGTRTLVLSHGFGFDQSVWHYLIPYLACYFKVVVFDLIFVNPNLYDPKKYSNFDSYAQDLVCLLDQLNVKKTIYLGHSMSAMIGCIAATKRPDLFEHLILLGGSPRYLNAEGYYGGFERSDIDKIFEAINENFPVWVQNFVPMAVGINNSAAIAEFEYSLGRMKPEIVLSVAKTVFLSDLRLVLPQVKVPCTIIQSREDIVAPTFIACYMKENLGDDATVKILETQGHFPQLTAFPLLLDALNQVLSIP*

>VvD14b

MLVIEKGLSAAMNARMIGFGNEAIVLAHGFGGDQSLWDKITPHLARSYRVLVFDWNFSGAVKDPSLYDSTKYSSYDAFADDLIALLDEFKLLASVFVGHSMSGMIGCIASIKRPELFKRLIFIAASPRYLNANNYEGGFERSEIEQIFANIESDFDKWASNFAPLAVDVNDPLSVEKVEKCIRRMRPEVALPLAKTVFCCDHRDILDKVTTPCTIVQPTNDIVAPISVAEYMQKKIKGKTTVEIIDMDGHFPQLTAHLQLLSVLDSVLVLSPDHQEK*

>VvD14c

MGNTLLEALNVRVVGNGERVLVLAHGFGTDQSAWQRILPYFLPHFRIILYDLVCAGSVNPDYFDFRRYTTLDAFVDDLLNILDALGVDRCAYVGHSVSAMIGILASIRRPELFTKLVLIGASPRFLNDHDYHGGFEEGEIEKVFSAMEANYDAWVHGFAPLSVGADVPAAVREFSRTLFNMRPDITLFVSRTIFNSDLRGVLGLVKVPCCIIQTAKDVSVPTSVALYLKNHLGGRNTVEMLNVEGHLPHLSAPMLLAPVLRRALSR*

>VvD14d

MKQYQIINISTTLFLSLLLHFLFFYQAMNARIIGSGNEAIVLAHGYGADQSFWDKITPSLARTYRVLVFDWNFSGSVKDPNLYDSAKYSSYDAFADDLIALLVEFNLRASVFMGHSMSGMIGCIASIKRPELFKRLILIGSSPRYFNDDNYEGGFESSVIEQMFSNMESNFDEWASYFASLVANAKNPLSVEKYEKSLRAMRPEVALSVAKTVFHCDERDILDKVMTPCTIIQTTNDAAVPNSVAEYMQKKIKGETTVEKIDMDGHFPHLNAHLQFLNVLGSVLGFNPHLESSDN*

>VvD14e

MGFILCFYDYEKSLRTIRPKVALSVAERVFCCDQRDILAKVMTPCTIIQPANDAAIPNSVAEYMQTKIKGETTVEKIDMDGHFPHLDAHLQFLNVLGSVLGFNPHLESSSN*

>VvLOB

MASSSSYNSPCAACKFLRRKCMPGCIFAPYFPPEEPQKFANVHKIFGASNVTKLLNELLPHQREDAVNSLAYEAEARVRDPVYGCVGAISFLQRQVERLQKELDAANADLIRYACNEMSSQLPSPSLVRSTSRRIGNEGGGSYFQNPGYSHPYSLPWDDNPSGNMNESGGGGGGGSM*

>VvLBD19

MTGSKGDGGGPCGACKFLRRKCVKGCVFAPYFDSDQGTAHFAAVHKVFGASNASKLLTGIPAHKRLDAVVTLCYEALARLRDPVYGCVAHVFTLQQQVMNLQAELAFIQARLSTLQRIPSLAPPPLLPESAPQPNLQSDFFNVPLQSHEASMEAADFCNNSMDRELEDGDLQTLAQEFVSRFLPGVKVRDSSSN*

>AtMAX2

MASTTLSDLPDVILSTISSLVSDSRARNSLSLVSHKFLALERSTRSHLTIRGNARDLSLVPDCFRSISHLDLSFLSPWGHTLLASLPIDHQNLLALRLKFCFPFVESLNVYTRSPSSLELLLPQWPRIRHIKLLRWHQRASQIPTGGDFVPIFEHCGGFLESLDLSNFYHWTEDLPPVLLRYADVAARLTRLDLLTASFTEGYKSSEIVSITKSCPNLKTFRVACTFDPRYFEFVGDETLSAVATSSPKLTLLHMVDTASLANPRAIPGTEAGDSAVTAGTLIEVFSGLPNLEELVLDVGKDVKHSGVALEALNSKCKKLRVLKLGQFQGVCSATEWRRLDGVALCGGLQSLSIKNSGDLTDMGLVAIGRGCCKLTTFEIQGCENVTVDGLRTMVSLRSKTLTDVRISCCKNLDTAASLKAIEPICDRIKRLHIDCVWSGSEDEEVEGRVETSEADHEEEDDGYERSQKRCKYSFEEEHCSTSDVNGFCSEDRVWEKLEYLSLWINVGEFLTPLPMTGLDDCPNLEEIRIKIEGDCRGKRRPAEPEFGLSCLALYPKLSKMQLDCGDTIGFALTAPPMQMDLSLWERFFLTGIGSLSLSELDYWPPQDRDVNQRSLSLPGAGLLQECLTLRKLFIHGTAHEHFMNFLLRIPNLRDVQLRADYYPAPENDMSTEMRVGSCSRFEDQLNSRNIID*

>AtSMXL6

MPTPVTTARECLTEEAARALDDAVVVARRRSHAQTTSLHAVSALLAMPSSILREVCVSRAARSVPYSSRLQFRALELCVGVSLDRLPSSKSPATEEDPPVSNSLMAAIKRSQANQRRHPESYHLQQIHASNNGGGGCQTTVLKVELKYFILSILDDPIVNRVFGEAGFRSSEIKLDVLHPPVTQLSSRFSRGRCPPLFLCNLPNSDPNREFPFSGSSGFDENSRRIGEVLGRKDKKNPLLIGNCANEALKTFTDSINSGKLGFLQMDISGLSLISIEKEISEILADGSKNEEEIRMKVDDLGRTVEQSGSKSGIVLNLGELKVLTSEANAALEILVSKLSDLLKHESKQLSFIGCVSSNETYTKLIDRFPTIEKDWDLHVLPITASTKPSTQGVYPKSSLMGSFVPFGGFFSSTSNFRVPLSSTVNQTLSRCHLCNEKYLQEVAAVLKAGSSLSLADKCSEKLAPWLRAIETKEDKGITGSSKALDDANTSASQTAALQKKWDNICQSIHHTPAFPKLGFQSVSPQFPVQTEKSVRTPTSYLETPKLLNPPISKPKPMEDLTASVTNRTVSLPLSCVTTDFGLGVIYASKNQESKTTREKPMLVTLNSSLEHTYQKDFKSLREILSRKVAWQTEAVNAISQIICGCKTDSTRRNQASGIWLALLGPDKVGKKKVAMTLSEVFFGGKVNYICVDFGAEHCSLDDKFRGKTVVDYVTGELSRKPHSVVLLENVEKAEFPDQMRLSEAVSTGKIRDLHGRVISMKNVIVVVTSGIAKDNATDHVIKPVKFPEEQVLSARSWKLQIKLGDATKFGVNKRKYELETAQRAVKVQRSYLDLNLPVNETEFSPDHEAEDRDAWFDEFIEKVDGKVTFKPVDFDELAKNIQEKIGSHFERCFGSETHLELDKEVILQILAASWSSLSSGEEEGRTIVDQWMQTVLARSFAEAKQKYGSNPMLGVKLVASSSGLASGVELPAKVDVIW*
