# Supplementary material for: VvD14c-VvMAX2-VvLOB/VvLBD19 module is involved in the strigolactone-mediated regulation of grapevine root architecture
Source: Mol Hortic. 2024 Oct 25;4:40. doi: 10.1186/s43897-024-00117-z (PMC11515387; doi:10.1186/s43897-024-00117-z)
Supplement: Supplementary file 2 — Additional File 2. Table S1. Primer sequences used for quantitative reverse transcription polymerase chain reaction (qRT-PCR). Table S2. Primer sequences for gene cloning. Table S3. Protein sequences used in this study. Table S4. Primer sequences used for vector construction [file 43897_2024_117_MOESM2_ESM.zip › Additional file 2 Table S4.docx]

**Table S4 Sequence of primers used for vector construction in this study.**

| **Gene Name** | **Forward primer sequences (5’→3’)** | **Reverse primer sequences (5’→3’)** | |
| --- | --- | --- | --- |
| *VvD14c-OE-GFP* | ACCAGTCTCTCTCTCAAGCTTATGGGTGACCTGTTAGAA | GCCCTTGCTCACCATGGATCCTCATCTCGACAGGGCTCGC | |
| *VvD14c-GFP* | ACCAGTCTCTCTCTCAAGCTTATGGGTGACCTGTTAGAA | GCCCTTGCTCACCATGGATCCTCTCGACAGGGCTCG | |
| *VvMAX2-AD* | GTGGGCATCGATACGGGATCCATGGCCGGAGCTGCTGC | CAGCTCGAGCTCGATGGATCCTCAATCAAGTATCCTCCG | |
| *VvD14c-BD* | TCAGAGGAGGACCTGCATATGATGGGTGACCTGTTAGAAG | TCGACGGATCCCCGGGAATTCTCATCTCGACAGGGCTCGC | |
| *VvMAX2-pXY106* | GGACGCCGGCGGATCCATGGCCGGAGCTGCTGC | AGCTCTGCAGGTCGACATCAAGTATCCTCCGCC | |
| *VvD14c-pXY104* | AGGTACCCGGGGATCCATGGGTGACCTGTTAGAAG | TGCCACCGCCGTCGACTCTCGACAGGGCTCGCC | |
| *VvMAX2-nluc* | AGCTCGGTACCCGGGATCCATGGCCGGAGCTGCTGCC | GTCCATTTGTTGGATCCATCAAGTATCCTCCGCCT | |
| *VvD14c-cluc* | GGCGGTACCCGGGATCCATGGGTAACACCCTCT | GTCCATTTGTTGGATCCCCGTGAGAGGGCACGACGG | |
| *VvMAX2-OE-GFP* | ACCAGTCTCTCTCTCAAGCTTATGGCCGGAGCTGCTGCC | GCCCTTGCTCACCATGGATCCTCAATCAAGTATCCTC | |
| *VvMAX2-GFP* | ACCAGTCTCTCTCTCAAGCTTATGGCCGGAGCTGCTGCC | GCCCTTGCTCACCATGGATCCATCAAGTATCCTCCGCC | |
| *VvMAX2-BD* | GTGGGCATCGATACGGGATCCATGGCCGGAGCTGCTGCC | CCGCTGCAGGTCGACGGATCCTCAATCAAGTATCCTCCG | |
| *VvLOB-AD* | TCAGAGGAGGACCTGCATATGATGGCTTCATCCAGCTCTTACA | CAGCTCGAGCTCGATGGATCCTCACATACTGCCTCCCCCTCCT | |
| *VvLBD19-AD* | TCAGAGGAGGACCTGCATATGATGACTGGAAGCAAGGGAGAT | CAGCTCGAGCTCGATGGATCCTTAGTTAGAACTCGAATCCCG | |
| *VvMAX2-pXY104* | AGGTACCCGGGGATCCATGGCCGGAGCTGCTGCC | TGCCACCGCCGTCGACATCAAGTATCCTCCGCCT | |
| *VvLOB- pXY106* | GGACGCCGGCGGATCCATGGCTTCATCCAGCTCTTACA | AGCTCTGCAGGTCGACCATACTGCCTCCCCCTCCTCC | |
| *VvLBD19- pXY106* | GGACGCCGGCGGATCC ATGACTGGAAGCAAGGGAGAT | AGCTCTGCAGGTCGACGTTAGAACTCGAATCCCGGAC | |
| *VvLOB-cluc* | GGCGGTACCCGGGATCCATGGCTTCATCCAGCTCTT | GTCCATTTGTTGGATCCCATACTGCCTCCCCCTC | |
| *VvLBD19-cluc* | GGCGGTACCCGGGATCCATGACTGGAAGCAAGGGAGA | GTCCATTTGTTGGATCCGTTAGAACTCGAATCCCGGA | |
| *VvLOB-OE-GFP* | CTCAAGCTTGGATCCATGGCTTCATCCAGCTCTTACA | GCTCACCATACTAGTTCACATACTGCCTCCCCCTCCT |  |
| *VvLBD19-OE-GFP* | CTCAAGCTTGGATCCATGACTGGAAGCAAGGGAGAT | GCTCACCATACTAGTTTAGTTAGAACTCGAATCCCG | |
| *VvLOB-GFP* | CTCAAGCTTGGATCCATGGCTTCATCCAGCTCTTACA | GCTCACCATACTAGTCATACTGCCTCCCCCTCCTCC | |
| *VvLBD19-GFP* | CTCAAGCTTGGATCCATGACTGGAAGCAAGGGAGAT | GCTCACCATACTAGTGTTAGAACTCGAATCCCGGAC | |
| *VvD14c-28b* | GCCATGGCTGATATCGGATCCGAATTCATGGGTAACACCCTCTTG | TCGAGTGCGGCCGCAAGCTTGTCGACGTCACCGTGAGAGGGCACGACG | |
| *pET28b* | TACGACTCACTATAGGGGAATTG | CTGAGCAATAACTAGCATAACCC | |
| *CFP79A* |  | GTTTACGTCGCCGTCCAGC | |
| *AD* | TAATACGACTCACTATAGGGCGA | TTTTCGTTTTAAAACCTAAGAGTC | |
| *BD* | TAATACGACTCACTATAGGGCGA | AGATGGTGCACGATGCACAG | |
| *nluc* | GATGACGCACAATCCCA | AGGGCGTATCTCTTCAT | |
| *cluc* | CCAAGAAGGGCGGAAAGATC | GTGTGCGCAATGAAACTGATG | |
| *pXY104* |  | CTTCTCGTTGGGGTCTTTGCT | |
| *pXY106* | GCGACACCCTGGTGAACCG | TCCATTTCACAGTTCGATAGCGA | |
